# Gene set enrichment analysis of RNA-Seq data: integrating differential expression and splicing

**DOI:** 10.1186/1471-2105-14-S5-S16

**Published:** 2013-04-10

**Authors:** Xi Wang, Murray J Cairns

**Affiliations:** 1 School of Biomedical Sciences and Pharmacy, The University of Newcastle, Callaghan, New South Wales, Australia; 2 Hunter Medical Research Institute, New Lambton, New South Wales, Australia; 3 Schizophrenia Research Institute, Sydney, New South Wales, Australia

## Abstract

**Background:**

RNA-Seq has become a key technology in transcriptome studies because it can quantify overall expression levels and the degree of alternative splicing for each gene simultaneously. To interpret high-throughout transcriptome profiling data, functional enrichment analysis is critical. However, existing functional analysis methods can only account for differential expression, leaving differential splicing out altogether.

**Results:**

In this work, we present a novel approach to derive biological insight by integrating differential expression and splicing from RNA-Seq data with functional gene set analysis. This approach designated SeqGSEA, uses count data modelling with negative binomial distributions to first score differential expression and splicing in each gene, respectively, followed by two strategies to combine the two scores for integrated gene set enrichment analysis. Method comparison results and biological insight analysis on an artificial data set and three real RNA-Seq data sets indicate that our approach outperforms alternative analysis pipelines and can detect biological meaningful gene sets with high confidence, and that it has the ability to determine if transcription or splicing is their predominant regulatory mechanism.

**Conclusions:**

By integrating differential expression and splicing, the proposed method SeqGSEA is particularly useful for efficiently translating RNA-Seq data to biological discoveries.

## Background

Transcriptome sequencing (RNA-Seq) is an increasingly important technology for transcriptome studies using the high-throughput sequencing (HTS) platforms. RNA-Seq reads can be used to measure overall expression levels by counting reads from each gene including different spliced isoforms collectively. More importantly the higher resolution can be used to detect transcript variants due to alternative splicing (AS), as well as alternative transcription start sites and alternative polyadenylation sites [1-4]. A number of recent studies have utilized RNA-Seq to quantify disease-associated transcriptome changes or discriminate between subtypes and help illuminate the molecular pathology of complex diseases at the RNA level (e.g., [5-8]).

In case-control transcriptome studies, identifying differentially expressed genes (DEGs) in a genome-wide scale is regarded as the first priority task since microarrays were applied to profile gene expressions. Existing DEG analysis tools dealing with RNA-Seq (such as DEGseq [9] and Cufflinks [10]) usually generate large lists of "interesting" genes or genome-scale gene ranks that can be used to generate new biological insight. The biological interpretation of DEGs has been assisted by computational functional analysis based on accumulated biological knowledge. This has culminated in databases such as the Kyoto Encyclopedia of Genes and Genomes (KEGG) [11] which aid in assembling the most enriched functional categories like pathways. According to the input type, functional enrichment tools can be categorized into two classes. The more traditional class is to take a list of preselected "interesting" genes, and applies statistical methods dealing with contingency tables to test the enrichment of each annotated gene set. The other class is reviewed as a cutoff-free strategy, which ranks all expressed genes according to the strength of expression difference, and adopts Kolmogorov-Smirnov-like tests to obtain enrichment significance. The cutoff-free strategy, which avoids choosing arbitrary cutoffs and can accumulate subtle expression changes of genes in the same set, has attracted a great deal of attention. Among them, Gene Set Enrichment Analysis (GSEA) [12] is a highly effective method, and has been successfully used in studying functional enrichment between two biological groups (e.g., [13]).

Originated for analysing microarray data, GSEA and its variants/extensions [12,14,15] take overall RNA abundance levels as a starting point, without regarding the differences between individual transcripts resulted from alternative splicing in genes. Evidence has shown that gene transcription and splicing take place simultaneously [16], and alternative splicing which occurs extensively [17] can substantially expand the variability of mRNAs from a limited number of genes in higher eukaryotes (e.g., in human [18]), leading to a polymorphism of protein structures and functions [19]. With RNA-Seq replacing microarrays, the ability to detect and quantify expression differences in individual transcripts after splicing, or equivalently the degree of alternative splicing in genes, is maturing. As an important regulatory mechanism in eukaryotes, alternative splicing should, and currently is able to, be taken into account in differential expression analysis. To the best of our knowledge, however, no tools till now have been able to integrate alternative splicing with each gene's overall expression for functional analysis.

Here we present a novel approach, named SeqGSEA, which first quantifies expression differences for each gene from exon read counts in two respects - overall expression and alternative splicing, and then combines the two respects for one integrated GSEA run. We modelled the read counts with negative binomial distributions, suitable for count data and capable of accounting for biological variation, and applied two strategies to integrate differential expression and splicing. Results on an artificial data set and real RNA-Seq data sets indicated that our approach can identify biologically meaningful gene sets through utilizing both overall expression and alternative splicing. Method comparison studies showed that the new approach outperformed other alternative pipelines for functional analysis of RNA-Seq data.

## Methods

### Basic assumptions

Gene expression at the RNA level can be recognized as either the total expression abundance of a gene regardless of the expression heterogeneity in individual isoforms or as individually expression proportion of discrete isoforms of a gene resulting from AS including the usage of alternative transcription start sites and alternative ploy(A) sites [20]. When comparing two groups of samples, we term the analysis regarding differential overall express of a gene, followed by microarray studies, as differential expression (DE), whilst the analysis regarding isoform proportion changes as differential splicing (DS). SeqGSEA integrates DE and DS of each gene from RNA-seq data to conduct gene set enrichment analysis.

Currently there are two main strategies for accounting AS from RNA-seq data: exon-centroid and isoform-centroid [21]. In short, the exon-centroid methods are based on exon expression levels, transforming the problem to detecting differential exon usage. In contrast, the isoform-centroid methods infer individual splice-isoform expression proportions in each gene first, and then quantify changes of the isoform expression between samples. To avoid introducing extra noises or biases in isoform expression level inference, SeqGSEA presented here quantifies AS between biological groups in an exon-centroid fashion.

Exon-centroid methods require read counts on each exon, of which the sum in a gene is exactly the input for computing overall expression changes. Known that there are at least seven types of AS [20] including alternative 3'/5' splice sites, therefore read counts based on the biological exon definition will, however, not sensitive to those types. In this approach, we define sub-exons as non-overlapping continuous exon fragments due to any possible splice sites (Supplementary Figure S1 in Supplementary Materials; all Supplementary Materials are in Additional file 1). Let $X_{ij}^{\left(g\right)}$ denote read counts on sub-exon *i *(*i = *1,2,..., *N*^(*g*)^) in gene *g *(*g *= 1,2,...,*G*) of sample *j *(*j = *1,2,..., *M *). By summing up the read counts of all sub-exons in a gene, the read count of gene *g *can be reached, denoted by $Y_{j}^{\left(g\right)},i.e.,Y_{j}^{\left(g\right)}=\sum_{i=1}^{N^{\left(g\right)}}X_{ij}^{\left(g\right)}$. Thus, differential gene expression analysis is divided into two sub-questions: DE analysis using gene read counts $Y_{j}^{\left(g\right)}$, and DS analysis based on sub-exon read counts $X_{ij}^{\left(g\right)}$ in gene *g *given the total count $Y_{j}^{\left(g\right)}$.

Over-dispersion is frequently observed in modeling read counts from RNA-Seq due to the non-uniform read distribution [22]. To solve this problem and account for biological variability when comparing groups of biological subjects, negative binomial (NB) distributions have been proposed to model the count data [23-26]. The NB distribution can be written in various forms with parameters, but those can be uniquely determined by its mean *μ *and variance *σ*^2^, as NB(*μ*, *σ*^2^). The mean parameter *μ *is the expectation value of the observed counts, while the variance parameter *σ*^2 ^includes a dispersion term, written as *σ*^2 ^= *μ + φμ*^2^, where *φ *is the dispersion parameter.

### DE and DS scores

Based on our modeling assumptions, we further borrow the idea from Anders and Huber's work [25] and our previous work [27] to derive DE and DS scores, representing the degree of differential overall expression and alternative splicing in each gene, respectively. Basically, a DE or DS score is a statistic in the form of squared difference of parameter estimates, divided by the sum of parameter variances. Note that by definition the DE or DS scores are all of non-negative values, and therefore so are gene scores (defined in the following sub-section), which makes it unable to tell a gene is exactly up or down regulated in the studied group. Two major reasons are considered to ignore the direction of expression changes. First, DS itself is of no directions; it will be meaningless to integrate one directed score with an undirected one. Second, it is also reasonable to consider only the absolute overall expression changes regardless of the direction. In biological pathways, reciprocal genes, such as those involved in feedback loops, are usually inversely regulated [15,28]. Taking both the up- and down-regulated components together would therefore reduce false-negatives that can occur in methodologies that consider the regulation direction.

Using NB distributions to model $Y_{j}^{\left(g\right)}$, we can write $Y_{j}^{\left(g\right)}∼\;\text{NB}(\mu_{gj},\;\sigma_{gj}^{2})$, where *μ_gj _*= *s_j_q_g_*_,*ρ*__(*j*). _That is, the mean parameter *μ_gj _*is the product of a size factor *s_j _*indicating the sequencing depth for sample *j*, and *q_g_*_,*ρ*__(*j*)_, which is proportional to the expectation value for gene *g *in group *ρ*(*j*). To measure the overall expression changes between group *A *and group *B*, we define the DE score for gene *g *as

(1) $$S_{DE}^{\left(g\right)}=\frac{{\left({\hat{q}}_{g,A}-{\hat{q}}_{g,B}\right)}^{2}}{\hat{V}(q_{g,A})+\hat{V}(q_{g,B})}$$

where ${\hat{q}}_{g,A}$ is the estimate of the expected expression $q_{g,A}$ of group *A*, and $\hat{V}\left(q_{g,A}\right)$ denotes the variance estimate of $q_{g,A}$; those with subscript B are for group *B*. The detailed derivation of parameter estimation can be found in Supplementary Note #1 in Supplementary Materials. Note that in the procedure, we use only the samples from one group (without information sharing across groups) to estimate the dispersion parameters. This is because SeqGSEA requires a moderate number of replicates in each group for the purpose of permutation, so that the per-group data could be enough to get stable estimates.

Similarly, we can define DS scores from sub-exon read counts as an average value across all sub-exons in a gene, i.e., for gene *g*

(2) $$S_{\text{DS}}^{\left(g\right)}=\frac{1}{N^{\left(g\right)}}\sum_{i=1}^{N^{\left(g\right)}}\frac{{\left({\hat{p}}_{i,A}^{\left(g\right)}-{\hat{p}}_{i,B}^{\left(g\right)}\right)}^{2}}{\hat{V}\left(p_{i,A}^{\left(g\right)}\right)+\hat{V}\left(p_{i,B}^{\left(g\right)}\right)}$$

where ${\hat{p}}_{i,A}^{\left(g\right)}$ is the estimate of the expected read count fraction of sub-exon *i *in the group *A*, $\hat{V}\left(p_{i,A}^{\left(g\right)}\right)$is the variance estimate of ${\hat{p}}_{i,A}^{\left(g\right)}$, and *N*^(*g*) ^is the number of sub-exons in gene *g*. Please find detailed derivation of parameter estimations in Supplementary Note #2 in Supplementary Materials.

### Integrated gene scores

Based on the definition and calculation of DE and DS scores for each gene to quantify differential overall expression and alternative splicing, respectively, we intend to propose an integrated gene score $S^{\left(g\right)}$ to depict a gene's RNA abundance difference with regards to the both respects. As the two scores for one gene may not be fully comparable, we include a normalization step before computing the gene score. While the main GSEA algorithm preforms a sample-shuffling strategy to obtain statistical significance, we need also compute DE and DS scores on the permuted data sets. Therefore, the distribution of permutation DE (DS) scores offers an empirical background for $S_{DE}^{\left(g\right)}\left(S_{DS}^{\left(g\right)}\right)$. We take the values divided by the mean permutation scores for normalization.

(3) $$S_{\text{DE},\text{norm}}^{\left(g\right)}=S_{\text{DE}}^{\left(g\right)}/\bar{T_{\text{DE}}^{\left(g\right)}},S_{\text{DS},\text{norm}}^{\left(g\right)}=S_{\text{DS}}^{\left(g\right)}/\bar{T_{\text{DS}}^{\left(g\right)}}$$

where $\bar{T_{DE}^{\left(g\right)}}\left(\bar{T_{DS}^{\left(g\right)}}\right)$ is the mean DE (DS) score for gene *g *over all permutations. An exemplified plot for normalized DE (DS) scores is shown in Supplementary Figure S2a(b) in Supplementary Materials.

Two strategies were applied to integrate DE and DS scores into one per-gene scores, one of which is to take weighted sums and the other is a rank-based strategy. Linear combination is the simplest yet typically used way for weighted summation, which writes

(4) $$S_{g}=\alpha S_{\text{DE},\text{norm}}^{\left(g\right)}+\left(1-\alpha \right)S_{\text{DS},\text{norm}}^{\left(g\right)}$$

where *α *∈ [0,1] is the weight balancing the contribution from DE and DS; the larger *α *is, the more contribution from DE is applied. Two extreme cases (*α *= 0 or 1) make the integration degenerate to DE- or DS-only analysis. See Supplementary Figure S2c for an exemplified plot of the integrated scores. We also considered weighted quadratic combination of the two scores, but it behaved similar to the linear combination and as such we ignore the discussion of the difference between linear, quadratic and high-order combination in this study.

Although the strategy to take the weighted sums of the two scores intuitively makes sense, it does not take account of the fact that different genes with overall expression changes and splicing changes can work together to function in the same gene set. For example, gene *a *is involved substantial DE regulation without DS but gene *b *in the same functional gene set undergoes DS but rarely DE; the linear combination may average out the changes and cause this gene set with expression significantly regulated undetectable. To accounts for the inconsistent DE and DS regulation of genes in the same gene set, we take a rank-based strategy to integrate scores, which will automatically assign more weights to higher ranked DE or DS scores. First, we rank DE and DS scores in ascending order, respectively, denoting the ranks $\gamma_{\text{DE}}^{\left(g\right)}$ and $\gamma_{\text{DS}}^{\left(g\right)}$ for gene *g*. Then, the integrated score writes

(5) $$S_{g}=\left(\alpha \gamma_{\text{DE}}^{\left(g\right)}S_{\text{DE},\text{norm}}^{\left(g\right)}+\left(1-\alpha \right)\gamma_{\text{DS}}^{\left(g\right)}S_{\text{DS},\text{norm}}^{\left(g\right)}\right)/\left(\alpha \gamma_{\text{DE}}^{\left(g\right)}+\left(1-\alpha \right)\gamma_{\text{DS}}^{\left(g\right)}\right)$$

We keep *α *in the formula for a global tuning of DE and DS contribution, in addition to the data-adapted weights given by the ranks.

Overall, regardless of *α*, a higher gene score indicates that total transcript abundance, transcript composition, or combination of both, is altered more dramatically. In practice, one may need to examine many weights for tweaking the contribution from DE or DS, as no prior knowledge currently gives the true contributions of DE and DS in a particular gene set. However, as we discuss in the *Results *section, with a sufficient number of weights been specified in the analysis, the detected gene sets will be saturated.

We get gene scores on the permuted data sets in the same way for the weighted linear combination, but for the rank-based strategy in two different ways: using the same ranks got from the real DE/DS scores (in a global manner), or using permutation-specific ranks obtained from each permutation.

### Gene set enrichment analysis

Based on the integrative gene score defined above and the prototype of GSEA originated by Mootha *et al. *in 2003 [13], we can convert the input RNA-Seq count data into biological interpretations. The major merit of GSEA is that it does not rely on any arbitrarily predefined threshold to select "interesting" genes for functional analysis. This is very important in human disease research as the subjects of these studies are usually subject to much larger biological variation than more controlled conditions in cell lines or model organisms [29]. Due to the high level of heterogeneity of human samples, statistically significant DE or DS genes are not always detected. These problems are exemplified in high-throughput analysis of neuropathology of neuropsychiatric disorders like schizophrenia (see comparison with Cuffdiff in *Results*). Furthermore, *p*-values from various DE gene detection methods may not be comparable [30], which may cause the functional analysis results with those methods cannot be streamlined, consequently making prediction about the biological significance unreliable. The strategy of GSEA successfully avoids the effect of arbitrary cutoffs and can aggregate a composite of weak evidence to identify functional significance.

Rather than other functional analysis methods, the GSEA algorithm takes into account how each gene is associated with a phonotype of interest, i.e., in this study the gene scores representing the magnitude of overall expression and splicing alterations in the studied group. As a result, if a gene set containing a number of genes that have collectively high enough gene scores, the gene set will be identified. Given an *a priori *defined gene set, the algorithm will report a Kolmogorov-Smirnov-like statistic, called enrichment score (ES, Supplementary Figure S2d), with the corresponding significance level based on permutation tests (empirical *p*-values and FDRs controlling global false positives). We permute each sample's class labels 1,000 times to yield statistical significance throughout this study. Please see Supplementary Note #3 in the Supplementary Materials for more details with formulas rewritten using unified denotations in this study.

## Results and discussion

### Data used

*Data sets*. Three recently generated real RNA-Seq data sets and one artificial data set were used for evaluating the proposed method. The three real data sets include one cancer transcriptome study [31] (hereafter the cancer data), and two schizophrenia transcriptome studies in different human brain areas, dorsolateral prefrontal cortex (DLPFC or BA46) [7] and superior temporal gyrus (STG or BA22) [8]. The cancer data, downloaded from NCBI SRA[32] with accession number SRP002628, were generated with 20 prostate cancer samples and 10 matched benign samples by Illumina GAII, 22.2 million reads on average for each sample. The BA46 data contains 20 schizophrenia samples and 20 matched controls, and were yielded by SOLiDv4 with an average sequencing depth 135 million reads per sample. The BA46 data used in this study were mapped reads (against hg19) in BAM format obtained from the authors. The BA22 data [33] were generated by Illumina GAII platform, but for a smaller sample size (9 cases vs. 9 controls), with 28.2 million reads on average per sample. The cancer and BA22 FASTQ data were mapped to the human reference genome (hg19) by Tophat (v1.4.1). Sub-exon read counts were obtained using Python scripts based on HTSeq [34]. The artificial data set, with various layers of differences but for characterizing the proposed method, was comprised by 10 control samples from the cancer data and 9 normal samples from the BA22 data. See Table 1 for other related experiment statistics.

**Table 1:** Correlation coefficients of DE and DS scores and experiment statistics of the four data sets.

Data Set	**Corr. Coef**.	P-value	Sample Size	Platform	SE/PE	Strand Specific	Read Length	Frag. Size	Tissue
Artificial	0.23	0	10 v 9	GAII	-	-	-	-	-
Cancer	0.05	0	20 v 10	GAII	PE	No	36	200-300	Prostate
BA46	-0.007	0.17	20 v 20	SOLiD v4	SE	Yes	50	200-300	Brain (BA46)
BA22	-0.033	3.4e-9	9 v 9	GAII	SE	No	76	200-250	Brain (BA22)

*Gene sets*. Six categories of gene sets from MSigDB v3.1 [12,35] were used in this study, including positional gene sets (c1, n = 326), curated gene sets (c2, n = 3272; due to the large size of the new version and many overlapping gene sets from different resources, v3.0 used instead), motif gene sets (c3, n = 836), computational gene sets (c4, n = 858), GO gene sets (c5, n = 1454), and oncogenic signatures (c6, n = 189). Of those, two categories, c2 and c5, are mainly focused in this study to report SeqGSEA's performance.

### Correlation between DE and DS scores

To investigate the possibility of a global association between differential overall expression and differential splicing in the four data sets, we first investigated the correlation of DE and DS scores. The artificial data set was comprised of two RNA-Seq experiments with different tissue types and demographics, so non-housekeeping genes may be regulated by both transcription and splicing. Not surprisingly, we observed a Pearson's correlation coefficient of 0.23 on the artificial data set, which is significantly larger than 0 (*p*-value = 0, Table 1, Supplementary Figure S3). On the three real data sets, even if the correlation coefficient was significantly not equal to 0, the correlation coefficient was very close to 0, indicating the global associations between DE and DS in these disease-related experiments were very weak (if any), but varied in different diseases (Table 1, Supplementary Figures S4-S6). The weak to null association of DE and DS in disease-related transcriptomes is consisted with previous studies using exon arrays [36]. Nevertheless, we still observed a proportion of genes having both relatively high DE scores and high DS scores (Supplementary Figures S4-S6), suggesting they were subject to both DE and DS regulation simultaneously. This observation validates the main assumption of our approach, and therefore it is reasonable that the integration of DE and DS with linear combination for functional analysis would work.

Besides, we also observed that the correlation coefficient decreases with the increase of read length. This trend may imply that read length would to some degree affect analysis results of RNA-Seq data, but this hypothesis needs to be further validated in the near future when more disease-related RNA-Seq data sets are available.

### SeqGSEA performance summary and saturation analysis

Recalling the strategies to generate integrated gene scores, for simplicity, we let "Linear" denote the linear combination strategy, while "RankSp" and "RankGlb" denote the rank-based integrative strategy with permutation-specific normalization and global normalization, respectively.

As mentioned above, the artificial data set was comprised of samples from two tissue types with unmatched demographics. Thus, as expected, SeqGSEA detected quite a number of gene sets on the artificial data set (Table 2, Supplementary Table S1). More importantly, with integrative gene scores proposed in this work, much more gene sets were detected when comparing with DE-only results (Supplementary Table S1). Venn diagrams shown in Supplementary Figure S7 further demonstrate that even the union of the gene sets detected by DE- and DS-only GSEA cannot cover all detected gene sets using integrated gene scores with different weights.

**Table 2:** The number of significant gene sets on the four data sets at FDR 1% with linear combination strategy.

Dataset	GS	DS	0.1	0.2	0.3	0.4	0.5	0.6	0.7	0.8	0.9	DE
Artificial	c2	1550	982	1389	1656	1773	1757	1569	1247	696	261	64
	c5	647	326	479	681	766	747	675	558	354	158	66

Cancer	c2	4	3	4	10	14	16	16	16	14	12	12
	c5	11	11	20	14	13	9	6	2	2	1	1

BA46	c2	0	1	1	2	3	3	2	4	3	2	2
	c5	0	2	2	2	2	2	2	2	2	1	0

BA22	c2	0	0	0	0	0	0	0	0	0	0	0
	c5	0	0	0	0	1	1	1	1	0	0	0

For the full results, see Supplementary Tables S1-S4.

GS: gene set category; DS: DS-only GSEA; DE: DE-only GSEA; 0.1,...,0.9: weights *α*.

A number of significant gene sets were detected by SeqGSEA at FDR 1% on the three real data sets; presumably because of the small sample size of the BA22 cohort and apparent batch effect, none or only very few gene sets were designated significant at FDR 1% (Table 2, Supplementary Tables S2-S4). Comparing with DE- or DS-only GSEA, essentially, more gene sets were detected to be significant using the integrated gene scores, which is similar as that on the artificial data set. Moreover, some gene sets could not be detected without the integration of DE and DS (see Venn diagrams in Supplementary Figures S8-S9); two extreme cases were observed where significant gene sets could only be detected by integrated gene scores in c5 on BA46 and BA22 (Supplementary Tables S3 and S4).

When we observed the intersection of detected gene sets with different weights (Venn diagram cores), only the artificial data shared gene sets with weights spanning from 0 to 1 (Supplementary Figures S7-S9). These shared gene sets were detected by DE-only and DS-only GSEA, and GSEA with integrated DE and DS scores by any weights. This is because the artificial transcriptome study contained samples from different tissue types and with different demographics, and many genes were subject to dramatic DE and DS. In contrast, the disease-associated alteration of the real disease transcriptomes was not extensive enough, so only one or few GSEA runs among DE-only, DS-only, or with integrated gene score of a particular weight could work. This observation in some way coincided with the correlation between DE and DS scores on the four data sets described above - comparing with the real data sets, there were much more genes subject to significant DE and DS simultaneously in the artificial data set, causing the correlation coefficients sufficiently larger than 0.

We also compared the different integration strategies and found that SeqGSEA with rank-based combination detected more gene sets that linear combination on the artificial and the cancer data sets (Supplementary Tables S1 and S2); however, on the BA46 and the BA22 data, the results from the two strategies were comparable (Supplementary Tables S3 and S4). Detailed analysis showed that most of the detected gene sets with different combination strategies were overlapped. We also found that the weight played a particularly critical role on SeqGSEA's performance with both linear combination and rank-based combination strategies, indicating that the global tuning of DE and DS contribution was more effective than the data-adapted tuning.

**Figure 1:**
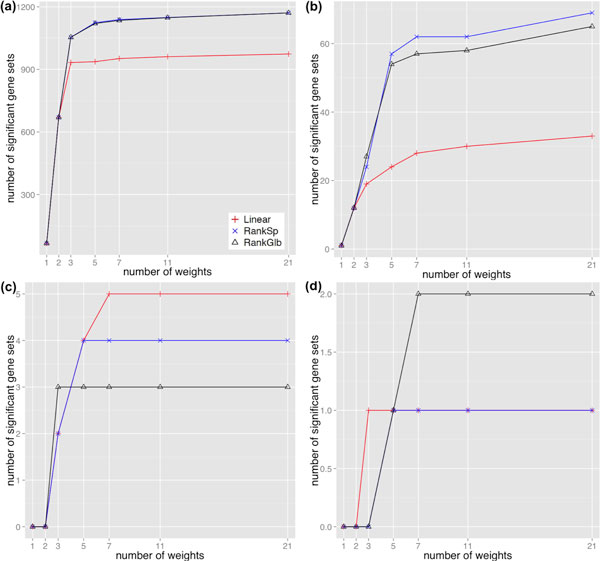
**Saturation plots of weights on gene set category c5**. Shown are the numbers of unique gene sets detected by different number of weights indicated in x-axis. (a) is for the artificial data sets; (b) cancer; (c) BA46; (d) BA22. From the fewest to the most number of weights, we gradually included the following weights in the order of (1 - DE-only, 0 - DS-only, 0.5, 0.1, 0.9, 0.3, 0.7, 0.2, 0.4, 0.6, 0.8, 0.05, 0.15, 0.25, 0.35, 0.45, 0.55, 0.65, 0.75, 0.85, 0.95). Red color indicates "Linear" combination, blue "RankSp", and black "RankGlb".

To optimize integration of DE and DS we found it necessary to explore a range of component weight *α *to investigate its effect on the integration efficiency. Clearly, the number of gene sets detected cannot be the only criterion, because the DE-only analysis still makes biological sense despite fewer reaching the threshold of statistical significance. While it would be desirable to test as many different weights as possible to obtain comprehensive exploration, we could not enumerate all weights between 0 and 1, as there are infinite possibilities. However, based on a saturation analysis achieved by gradually adding more weights to check the unique gene sets detected by each number of weights, we found that most of SeqGSEA saturated at 7 or 11 weights regardless of the integration strategies; in some circumstances there was an increase when 21 weights were applied, but the increased amount was either a quite small number or not comparable with the increase at the beginning (Figure 1 and Supplementary Figures S10-S14). Therefore, we suggest that in practice about 11 weights (say 0, 0.1, 0.2,..., and 1) could be enough, and all detected gene sets with the 11 weights should be taken to form a comprehensive result. A similar saturation analysis is also suggested to check whether the 11 weights are sufficient.

### Biological insights

SeqGSEA detected the majority of gene sets on the artificial data set (Table 2), while the remaining gene sets were believed to contain genes without sufficient collective expression/splicing changes. When we examined these undetected ones, we found that most of them were relevant to housekeeping functions, such as in c2: DNA replication, cell cycle, and basal transcription factors; in c5: cellular homeostasis and RNA elongation. This indicates that the SeqGSEA approach was able to detect overrepresented gene sets with reasonably high specificity.

A detailed analysis on the BA46 results showed that the detected gene sets are of high biological relevance. For example, one c5 gene set detected by SeqGSEA in common with α = 0.2-0.8 shows the regulation of angiogenesis is relevant to schizophrenia. It has been reported that the failure of angiogenesis damages neurogenesis, particularly in neural structure, and therefore the genes involved in angiogenesis may also be important for schizophrenia [37]. Interestingly, with a high weight on DS, two gene sets regarding taste perception were detected. Evidence also has shown that taste-blindness is highly associated with schizophrenia [38,39], but more importantly, our analysis suggested that this association was largely formulated through the regulation of alternative splicing. The full results on BA46 with analysis are available in Supplementary Table S5, and those for the cancer data are in Supplementary Tables S6-S7. These results showed that integrating DE and DS for functional analysis yielded biologically meaningful interpretation in disease transcriptome studies. This analysis also indicated that specific combination weights could potentially reveal corresponding predominant regulatory mechanisms in detected gene sets.

### Comparison with other alternative analysis pipelines

The development of SeqGSEA was motivated by the failure of Cuffdiff analysis to reveal altered genes in the two schizophrenia data sets (BA46 and BA22). Noted that Cuffdiff is still in a positive developing stage, we compared two versions of Cuffdiff on the three real data sets (see Supplementary Note #4 for running parameters). In general, the newest version 2.0.2 was more stringent than the older one v1.3.0. Cuffdiff version 2.0.2 didn't detect any genes that undergo DE or DS (including differential promoter usage) on the cancer and BA22 data sets. We also noticed that the new version was very time consuming, and it failed to finish running the BA46 data within the running time limit (200 hours) of our HPC server, even though eight cores were specified to use. Although Cuffdiff v1.3.0 was less stringent, it only detected one gene (TMIGD2) as DS on the cancer data, and one gene (VANGL1) with differential promoter usage on the BA46 data. On the BA22 data set, 6 genes were detected as DS. With the aid of IPA [40] for functional analysis, no canonical pathways was detected at *p*-value cutoff 0.05; and top biological functions given by IPA included functions related to cancer, carbohydrate metabolism, and endocrine system development and function, all of which have no obvious association with schizophrenia. To conclude, SeqGSEA was not only more powerful than the pipeline constructed by Cuffdiff and IPA, but also yielded results of more biological relevance.

Another possible pipeline could be the traditional DE-only GSEA, to which the gene expression levels of samples are fed. The gene expression levels estimated from RNA-Seq data were generally not well variance stabilized [41], so we took the degenerated SeqGSEA results when *α *= 1 as the DE-only results. The result comparison and the advantage of SeqGSEA over DE-only have been described previously. Apparently, either DE-only or DE-only plus DS-only GSEA is not sufficient to detect all function-related gene sets. Once again, we suggest the functional analysis should consider the integration of DE and DS, which facilitates revealing overrepresented gene sets in disease transcriptomes more comprehensively and in a more biologically relevant manner, as it is clearly that a proportion of genes are subject to both DE and DS simultaneously.

## Conclusions

The method SeqGSEA proposed in this work is particularly useful for efficiently translating HTS transcriptome read data to biological discoveries, by integrating transcription and splicing, the two respects affecting gene expression at the RNA level, enhancing the discovery of overrepresented gene sets with combinatory transcript abundance changes. It is also beneficial to detect overrepresented gene sets with only major functional isoforms switched, where overall transcript abundance levels are unchanged. As a cutoff-free approach, SeqGSEA does not require any arbitrary criteria for selecting DE or DS genes, but generates more informative biological interpretation based on the powerful prototype of the GSEA method. With a linear combination strategy, SeqGSEA can potentially throw light on the regulatory preference of a particular set of genes over transcription regulation through, for example, transcription factors, or alternative splicing through splicing factors. SeqGSEA also provides a framework for integrating other gene-level information with transcriptome data, such as SNPs, for functional expression quantitative trait locus (eQTL) analysis.

SeqGSEA is particularly suitable for disease-related RNA-Seq studies, in which a moderate number of patient samples with matched controls are available. Most existing studies are in the order of ten subjects per group [5-8,42], with a few exceptions of large-scale sequencing studies with more than one hundred samples (e.g., [43]). The at least moderate sample size makes it possible for SeqGSEA to conduct its sample-randomization permutation strategy. Notably, a sufficient number of human individuals is vitally important to reach statistical significance and guarantee reproducibility because of the considerable biological variability, although HTS technologies have made the technical variation small [44]. Moreover, many human diseases display a hierarchical structure, with various subtypes that have not been completely recognized or resolved. Pooling samples together with different subtypes challenges DE/DS gene detection, but GSEA-like approaches can overcome this by directly considering biological insight at, for example, the pathway level, accumulating subtype-specific expression alterations in different pathogenic genes located in the same pathogenic pathway.

We have noticed covariates and batch effect involved in transcriptome studies and other unfavorable biases in RNA-Seq data may affect SeqGSEA's results. Sequencing biases can be adjusted by properly modeling read distributions (e.g., [45]); batch effects and effects from other covariates including RNA integrity and demographic factors can be identified and regressed in future development of the methodology. Further work will also provide a basis for reducing ambiguity in the integration strategies, which should enable the implementation of more sophisticated and facile approaches in future versions. The results reported in this study were only based on the Molecular Signatures Database (MSigDB), but in fact any sets of genes can be fed to SeqGSEA. With more high-throughput data converting to biology knowledge in the near future, curated gene sets will be added or improved, which will in turn yield more biologically significant results in functional analysis with the aid of computational tools like SeqGSEA.

## List of abbreviations

HTS: high-throughput sequencing; AS: alternative splicing; DEG: differentially expressed gene; GSEA: gene set enrichment analysis; NB: negative binomial; DE: differential expression; DS: differential splicing; FDR: false discovery rate.

## Competing interests

The authors declare that they have no competing interests.

## Authors' contributions

XW developed and briefly implemented the method, and analyzed the artificial and the real data. MC conceived of the study and participated in experiment design, result analysis and discussions. XW and MC drafted and revised the manuscript. Both authors read and approved the final manuscript.

## Supplementary Material

Additional file 1 **Supplementary Materials containing Supplementary Notes #1-4, Supplementary Figures S1-S14 and Supplementary Tables S1-S7**. Click here for file

## Declarations

The publication costs of this article were funded by Schizophrenia Research Institute, Sydney.

This article has been published as part of *BMC Bioinformatics *Volume 14 Supplement 5, 2013: Proceedings of the Third Annual RECOMB Satellite Workshop on Massively Parallel Sequencing (RECOMB-seq 2013). The full contents of the supplement are available online at http://www.biomedcentral.com/bmcbioinformatics/supplements/14/S5.
