# Supplementary material for: Gene set enrichment analysis of RNA-Seq data: integrating differential expression and splicing
Source: BMC Bioinformatics. 2013 Apr 10;14(Suppl 5):S16. doi: 10.1186/1471-2105-14-S5-S16 (PMC3622641; doi:10.1186/1471-2105-14-S5-S16)
Supplement: Additional file 1 — Supplementary Materials containing Supplementary Notes #1-4, Supplementary Figures S1-S14 and Supplementary Tables S1-S7. [file 1471-2105-14-S5-S16-S1.PDF]

Additional file 1 - Supplementary Materials for

Gene Set Enrichment Analysis of RNA-Seq Data: Integrating Differential Expression and Splicing

Xi Wang[1,2], Murray J. Cairns[1,2,3*]

[1]School of Biomedical Sciences and Pharmacy, The University of Newcastle, Callaghan, New South Wales, Australia
[2]Hunter Medical Research Institute, New Lambton, New South Wales, Australia
[3]Schizophrenia Research Institute, Sydney, New South Wales, Australia
*Corresponding author

Section I - Supplementary Notes

Supplementary Note #1: Differential overall expression

For differential overall expression analysis, the task is to quantify, for each gene, how large the total mRNA abundance difference is. With the assumption that the number of reads that mapped to gene *g* in sample *j* can be modelled by a negative binomial (NB) distribution, we can write

$$Y_j^{(g)} \sim \mathrm{NB}(\mu_{gj}, \sigma_{gj}^2) \tag{1}$$

When comparing two groups of samples (e.g., in case-control studies), we further assume that the mean parameter μ_{gj} is the product of a size factor s_j, indicating the sequencing depth in sample *j*, and $q_{g,\rho(j)}$, which is proportional to the expectation value for gene *g* in group $\rho(j)$, i.e.,

$$\mu_{gj} = s_j q_{g,\rho(j)} \tag{2}$$

Thus, the variance parameter can be expanded as,

$$\sigma_{gj}^2 = s_j q_{g,\rho(j)} + \phi_{g,\rho(j)} s_j^2 q_{g,\rho(j)}^2 \tag{3}$$

According to Equ. (2) and (3), there are one set of sample-specific parameters s_j, and two sets of group-specific parameters $q_{g,\rho(j)}$ and $\phi_{g,\rho(j)}$ to be estimated. Following Andres and Huber's analysis [1], to avoid the effect of a few extremely expressed genes, we take the median of the ratios of observed counts in one sample over a pseudo-reference sample, to estimate the size factor,

$$\hat{s}_j = \mathrm{median}_g \frac{Y_j^{(g)}}{\left(\prod_{k=1}^{M} Y_k^{(g)}\right)^{1/M}} \tag{4}$$

The denominator represents the pseudo sample by taking the geometric mean across samples. This strategy provides a more robust normalization on read counts across multiple samples than taking the total (or average) number of reads, such as RPKM. The newly released Cuffdiff 2 has also set this strategy rather than RPKM as default to normalize between RNA-Seq samples [2]. Similarly as what have been done in [1], we take the average of normalized read counts from the samples *j* belonging to the sample group, say *A*, to estimate $q_{g,A}$,

$$\hat{q}_{g,A} = \frac{1}{M_A} \sum_{j \in A} \frac{Y_j^{(g)}}{\hat{s}_j} \tag{5}$$

and the same way to estimate $\phi_{g,\rho(j)}$,

$$\hat{\phi}_{g,A} = \frac{\frac{1}{M_A - 1} \sum_{j \in A} \left(\frac{Y_j^{(g)}}{\hat{s}_j} - \hat{q}_{g,A} \right)^2 - \frac{\hat{q}_{g,A}}{M_A} \sum_{j \in A} \frac{1}{\hat{s}_j}}{\hat{q}_{g,A}^2} \tag{6}$$

where M_A is the number of samples in group A. Assuming $Y_j^{(g)}$ are independent variables and neglecting the difference between the true size factors s_j and their estimates $\hat{s}_j$, we can estimate the variance of $\hat{q}_{g,A}$ by

$$\hat{V}(q_{g,A}) = \frac{1}{M_A}\left(\frac{\hat{q}_{g,A}}{M_A}\sum_{j\in A}\frac{1}{\hat{s}_j} + \hat{\phi}_{g,A}\hat{q}_{g,A}^2\right) \tag{7}$$

Once the estimation done, we can easily define a per-gene score quantifying the total expression difference between two groups,

$$S_{\mathrm{DE}}^{(g)} = \frac{\left(\hat{q}_{g,A}-\hat{q}_{g,B}\right)^2}{\hat{V}(q_{g,A})+\hat{V}(q_{g,B})} \tag{8}$$

where $\hat{q}_{g,A}$ is the estimate of the expected expression $q_{g,A}$ of group A, and $\hat{V}(q_{g,A})$ denotes the variance estimate of $q_{g,A}$; those with subscript B are for group B.

Note that we estimate $\phi_{g,\rho(j)}$ using only the samples from one group without information sharing across groups. This is because SeqGSEA requires a moderate number of replicates in each group for the purpose of permutation, so that the per-group data could be enough to get stable estimates.

Supplementary Note #2: Differential alternative splicing

Intuitively, differential alternative splicing can be assessed through comparing the expression proportions of component isoforms in one gene, known as isoform-centroid methods, which however requires isoform expression inference beforehand. On the other hand, it has been shown that differential AS analysis can also be carried out by assessing each gene's sub-exon composition proportions [3]. This exon-centroid strategy is equivalent to the isoform-centroid one in most genes of the human genome [3], but has the deserved advantage that it can cut down the unnecessary computational demands and avoid extra uncertainty introduced during the inference process.

Following the NB distribution assumption for count data, we have

$$X_{ij} \sim \mathrm{NB}(\mu_{ij}, \sigma_{ij}^2) \tag{9}$$

From this formula we omit the superscript (g) for clarity, as we only consider one gene in the following derivation. In practice, genes are treated iteratively. The task for DS analysis is to compare the sub-exon read count vector $\boldsymbol{X}_{\cdot j}$ across sample groups conditional on this gene's total read count Y_j. We further assume that μ_{ij} is only proportional to Y_j and $p_{i,\rho(j)}$, the latter denoting the expected expression fraction of sub-exon i in the group $\rho(j)$ that sample j belongs to. So,

$$\mu_{ij} = Y_j p_{i,\rho(j)},\ \sigma_{ij}^2 = Y_j p_{i,\rho(j)} + \phi Y_j^2 p_{i,\rho(j)}^2 \tag{10}$$

Similarly as DE analysis, to define a DS score, we need to estimate $p_{i,\rho(j)}$ and its variance. To minimize the estimation variance, a weighting scheme is applied. Taking group A for example, we have

$$\hat{p}_{i,A} = \sum_{j=1}^{M_A} w_j \frac{X_{ij}}{Y_j} \tag{11}$$

with its variance

$$V(\hat{p}_{i,A}) = \sum_{j=1}^{M_A} w_j^2 \frac{\hat{p}_{i,A}+\phi Y_j \hat{p}_{i,A}^2}{Y_j} \tag{12}$$

where w_j>0 ($j = 1,2,\ldots,M_A$) denote the weights for samples with a constraint that $\sum_{j=1}^{M_A} w_j = 1$. It is easy to show that $\hat{p}_{i,A}$ is an unbiased estimator regardless of what values w_j take. To obtain the optimal weights w_j, we solve the following constrained optimization problem by using the Lagrange multiplier method,

$$\min \sum_{i=1}^{N} V(\hat{p}_{i,A}) = \sum_{i=1}^{N}\sum_{j=1}^{M_A} w_j^2 \frac{\hat{p}_{i,A}+\phi Y_j \hat{p}_{i,A}^2}{Y_j}, s.t. \sum_{j=1}^{M_A} w_j = 1 \tag{13}$$

and get

$$w_j \propto \frac{Y_j}{1+\phi Y_j \sum_{i=1}^{N} \hat{p}_{i,A}^2} \tag{14}$$

This indicates that more weights are put for samples with higher Y_j, due to higher overall expression or deeper sequencing. Next, we use Quasi-Likelihood (QL) method to estimate the dispersion parameter ϕ. We introduce an iteration procedure to update w_j and ϕ in turn to get converged solutions. Details can be found in our previous work [3].

Having all estimation done, we can also define a per-gene score to quantify the AS difference between two groups by averaging each sub-exon's differences.

$$S_{\mathrm{DS}}^{(g)} = \frac{1}{N^{(g)}} \sum_{i=1}^{N^{(g)}} \frac{\left(\hat{p}_{i,A}^{(g)} - \hat{p}_{i,B}^{(g)}\right)^2}{\hat{V}\left(p_{i,A}^{(g)}\right) + \hat{V}\left(p_{i,B}^{(g)}\right)} \tag{15}$$

Supplementary Note #3: Gene set enrichment analysis

Enrichment score (ES) is the main concept in GSEA. Given an *a priori* defined gene set *X*, the ES is a Kolmogorov-Smirnov-like statistic and reflects the degrees how the genes in *X* overrepresented at the top of the gene list sorted by gene scores in a descending order. To compute ES, we first rank gene scores decreasingly, forming an ordered gene list $L = \{g_1, g_2, \ldots, g_G\}$. By walking down the list, we introduce two vectors to assist ES computation: one is for the fraction of genes in *X* up to a position i in list *L*, weighted by a factor depending on their gene scores, denoted by $P_{\mathrm{in}}(X, i)$; the other for the fraction of genes not in *X*, denoted by $P_{\mathrm{out}}(X, i)$.

$$P_{\mathrm{in}}(X, i) = \sum_{g_j \in X, j \le i} \frac{S_{g_j}^p}{W}, \; P_{\mathrm{out}}(X, i) = \sum_{g_j \notin X, j \le i} \frac{1}{G - G_X} \tag{16}$$

where $W = \sum_{g_j \in X} S_{g_j}^p$, representing a normalization factor for genes in set *X*, while $(G - G_X)$ is for genes outside and G_X denotes the total number of genes in gene set *X*. Based on the two vectors, ES can be computed as (Supplementary Figure S1d)

$$E_X = \max_i [P_{\mathrm{in}}(X, i) - P_{\mathrm{out}}(X, i)] \tag{17}$$

We denote the position where the enrichment score is achieved as i_0, and the leading set X_0 is defined as the subset of *X* whose positions in list *L* not behind i_0. Notably, when $p = 0$, E_X reduces to the standard Kolmogorov-Smirnov statistic; when $p = 1$, genes in *X* are weighted just by their gene scores; when $p > 1$, genes in *X* with large gene scores will be weighted exponentially more. We set $p = 1$ for the analyses in this study.

To estimate the significance level of ES, we perform empirical permutation tests by shuffling samples' group labels. The permutation serves a null distribution for the observed ES, so the empirical *p*-values can be calculated according to this null distribution. For the adjustment of multiple hypothesis testing when multiple gene sets evaluated simultaneously, E_X are normalized and made comparable across the whole datasets of gene sets. Similar to the normalization of DE/DS scores, the normalized score $E_{X,\mathrm{norm}}$ equals to E_X divided by the mean value of permutation ES for the same gene set *X*. Then, *FDR* is defined as the ratio of the number of normalized permutation ESs exceeding $E_{X,\mathrm{norm}}$, to the number of normalized observed ESs no less than $E_{X,\mathrm{norm}}$. We set the number of permutations to be 1,000 to generate results throughout this study.

Supplementary Note #4: Cuffdiff parameters

All Cuffdiff results were based on RefSeq gene annotation, downloaded from UCSC website (http://hgdownload.cse.ucsc.edu/goldenPath/hg19/database/refFlat.txt.gz) and then converted to GTF format.

Cuffdiff parameters for analyzing the cancer data
Version 1.3.0: upper-quartile-norm, compatible-hits-norm, multi-read-correct, library-type=fr-unstranded, and others were in default setting.
Version 2.0.2: geometric-norm, compatible-hits-norm, no-effective-length-correction, multi-read-correct, library-type=fr-unstranded, and others were in default setting.

Cuffdiff parameters for analyzing the BA46 data
Version 1.3.0: upper-quartile-norm, compatible-hits-norm, multi-read-correct, library-type= fr-secondstrand, frag-len-mean=250, frag-len-std-dev=50, and others were in default setting
Version 2.0.2: running time exceeded the upper limit of our HPC server.

Cuffdiff parameters for analyzing the BA22 data
Version 1.3.0: upper-quartile-norm, compatible-hits-norm, multi-read-correct, library-type=fr-unstranded, frag-len-mean=225, frag-len-std-dev=25, and others were in default setting.
Version 2.0.2: geometric-norm, compatible-hits-norm, no-effective-length-correction, multi-read-correct, library-type=fr-unstranded, frag-len-mean=225, frag-len-std-dev=25, and others were in default setting.

Section II - Supplementary Figures

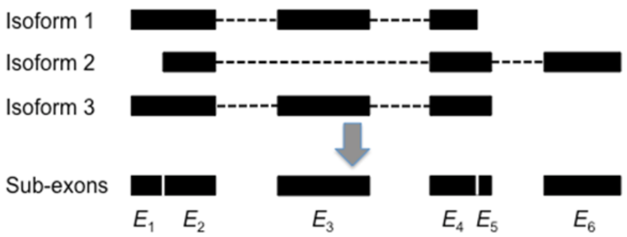

Supplementary Figure S1. Definition of sub-exons: non-overlapping continuous exon fragments separated by any possible splice sites.

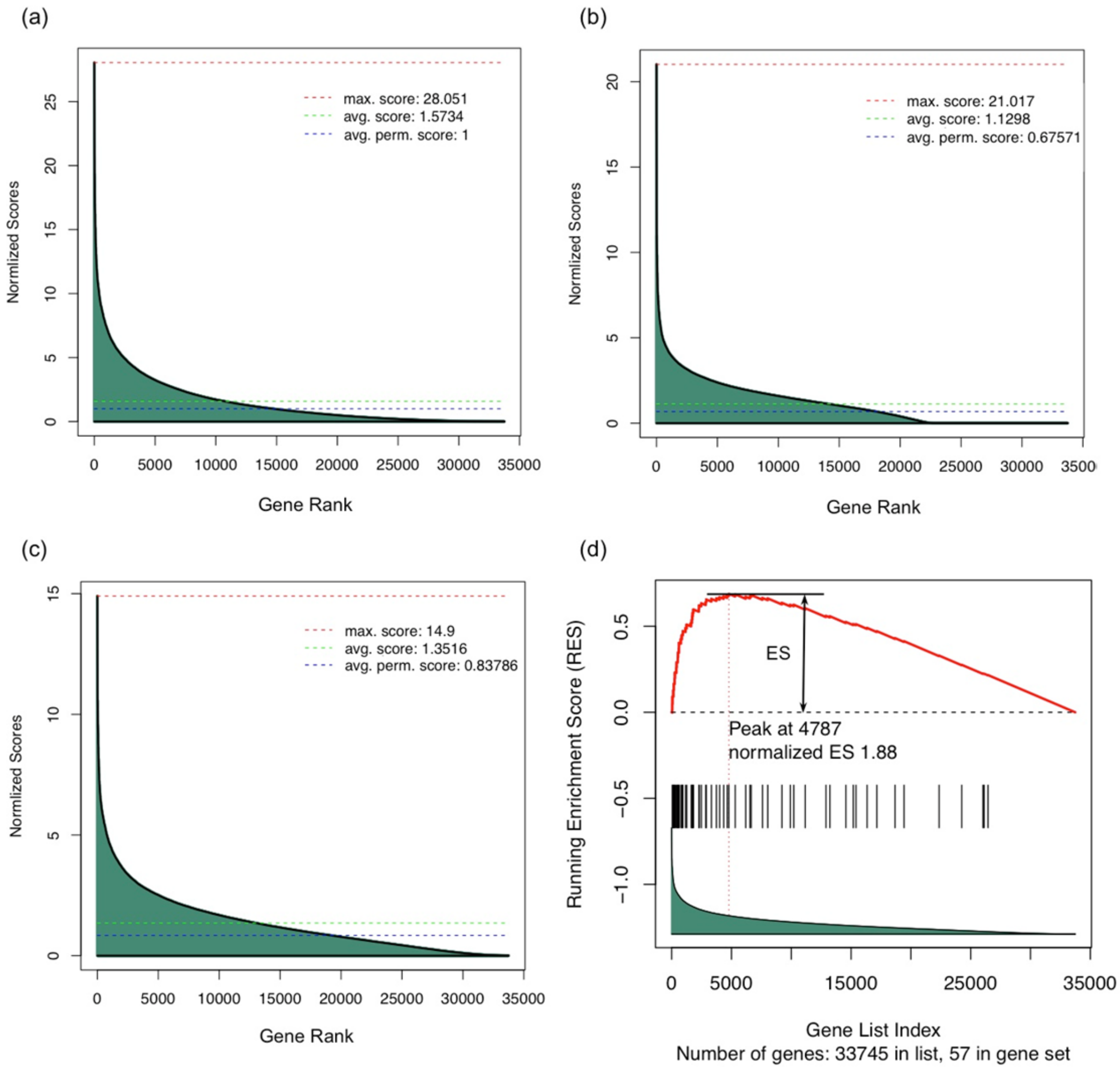

Supplementary Figure S2. Exemplified plots to demonstrate SeqGSEA's workflow. (a) Normalized DE scores, sorted from the largest to the smallest. (b) Normalized DS scores, sorted from the largest to the smallest. (c) Sorted gene scores with the weighting parameter alpha=0.5. (d) Plot of the enrichment score for an exemplified gene set. Top: running enrichment score, whose maximum is the enrichment score; middle: gene locations according to the sorted gene scores; bottom: sorted gene scores.

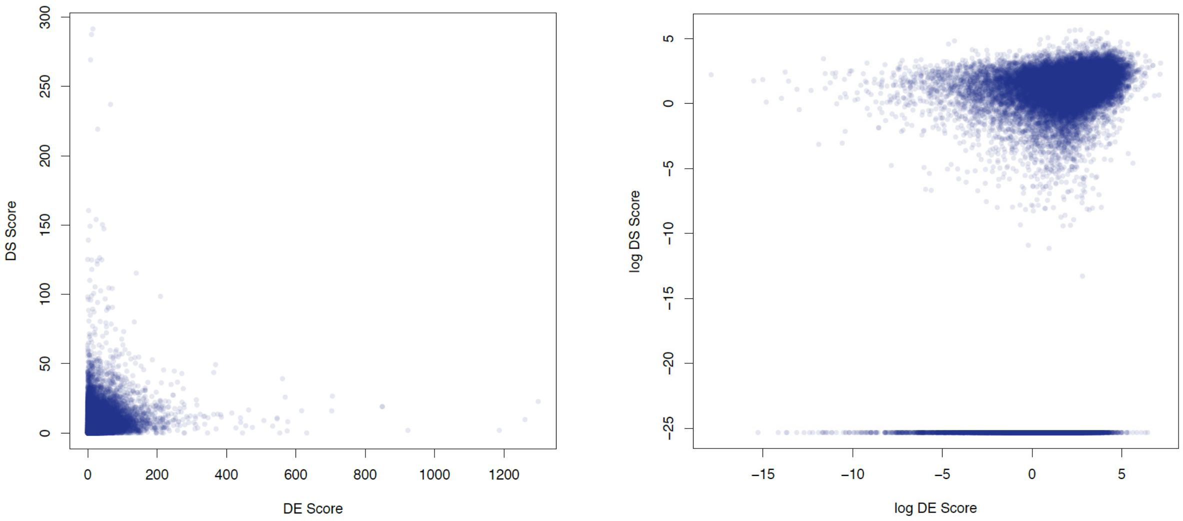

Supplementary Figure S3. Scattor plot of DE and DS scores on the artificial data set. Left: on the natural scale, correlation efficient 0.23, p-value 0; Right: on the log scale, correlation efficient 0.33, p-value 0.

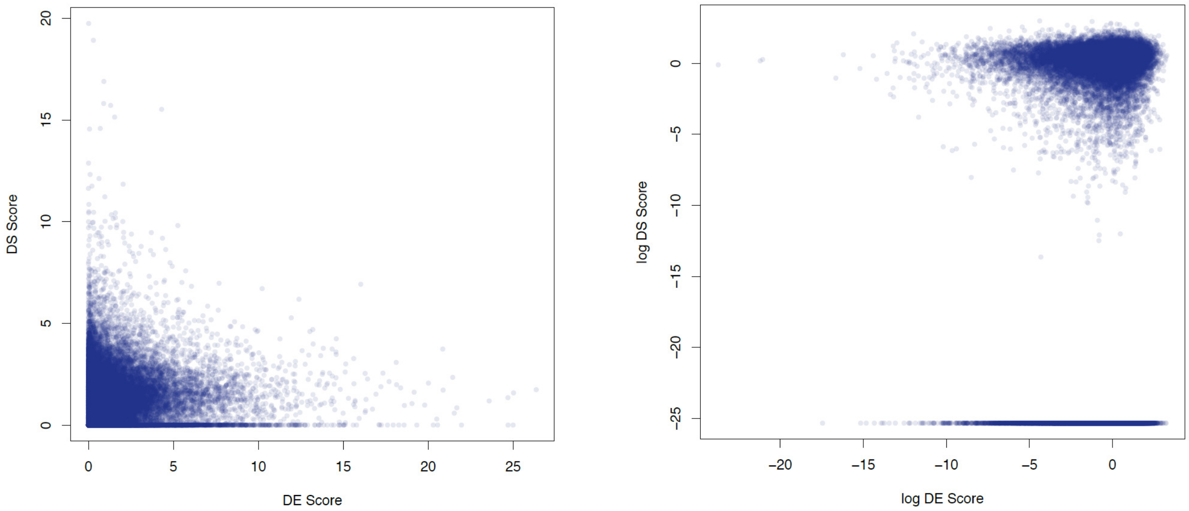

Supplementary Figure S4. Scattor plot of DE and DS scores on the cancer data set. Left: on the natural scale, correlation efficient 0.050, p-value 0; Right: on the log scale, correlation efficient 0.059, p-value 0.

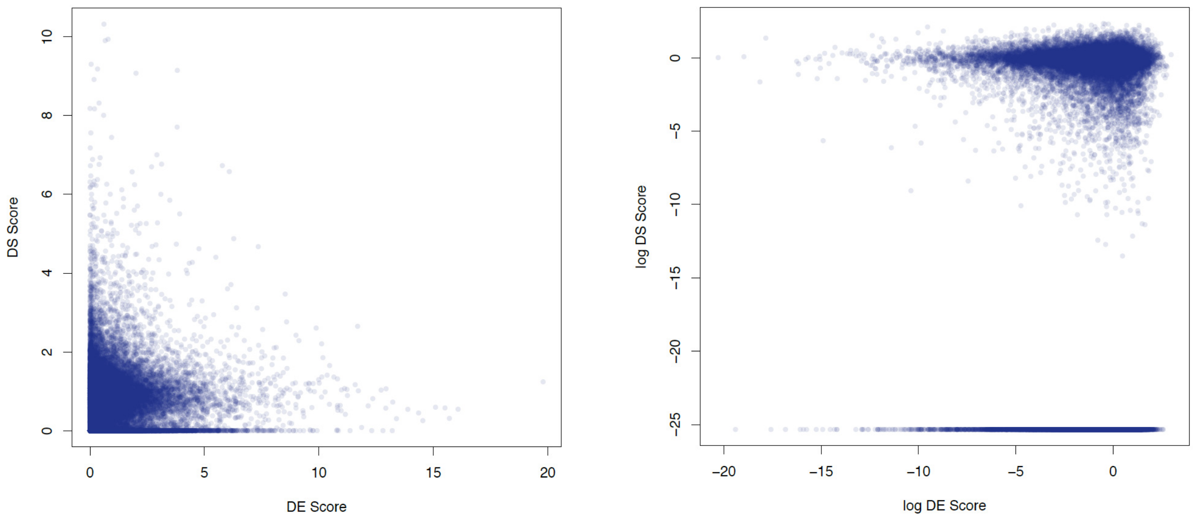

Supplementary Figure S5. Scatter plot of DE and DS scores on the BA46 data set. Left: on the natural scale, correlation efficient -0.0074, p-value 0.17; Right: on the log scale, correlation efficient -0.019, p-value 3.6e-4.

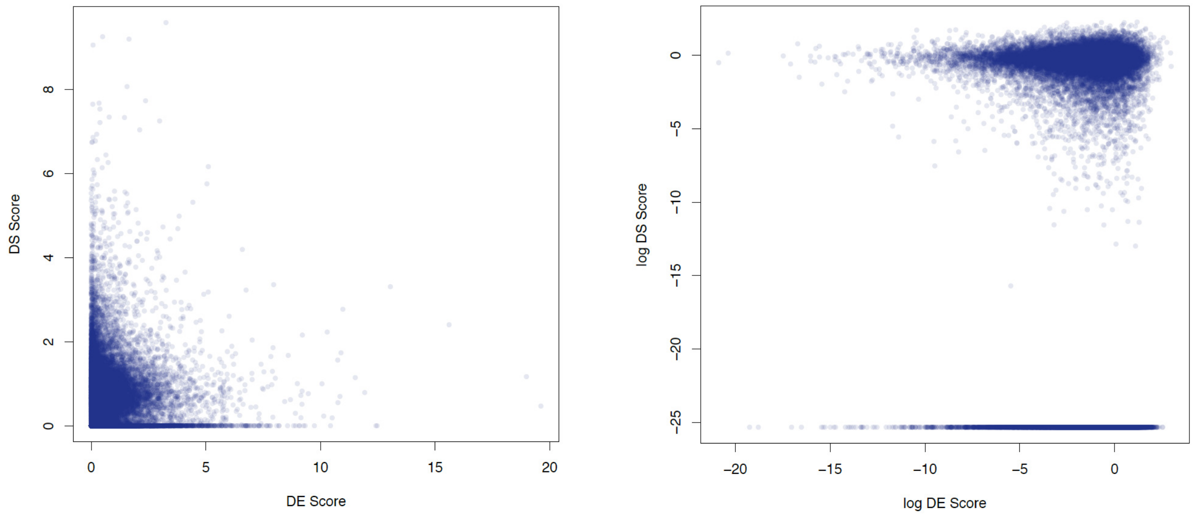

Supplementary Figure S6. Scatter plot of DE and DS scores on the BA22 data set. Left: on the natural scale, correlation efficient -0.033, p-value 3.4e-9; Right: on the log scale, correlation efficient -0.087, p-value 5.0e-55.

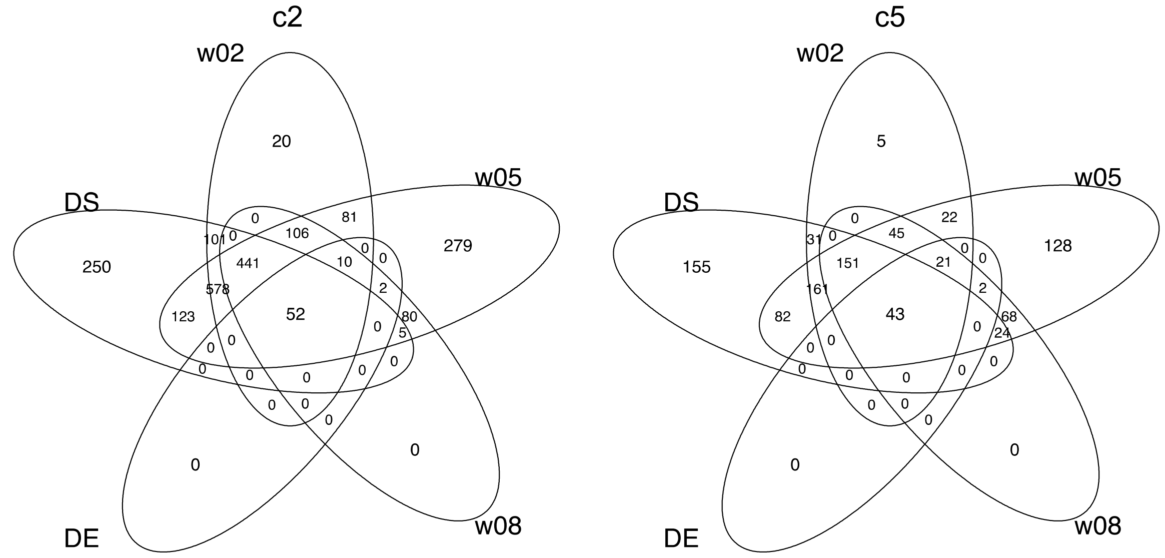

Supplementary Figure S7. Venn diagrams of significant c2 & c5 gene sets with different α on the artificial data set at FDR 1% with linear combination strategy. DS – DS-only GSEA; DE – DE-only GSEA; w01 – weight α=0.1 and so on so forth.

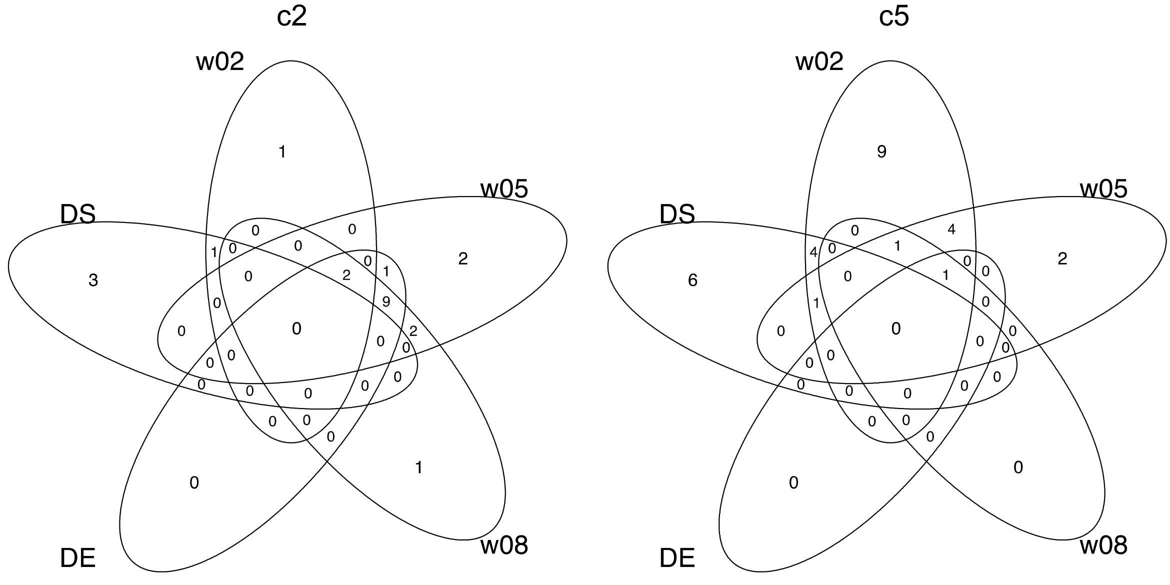

Supplementary Figure S8. Venn diagrams of significant c2 & c5 gene sets with different α on the cancer data set at FDR 1% with linear combination strategy. DS – DS-only GSEA; DE – DE-only GSEA; w01 – weight α=0.1 and so on so forth.

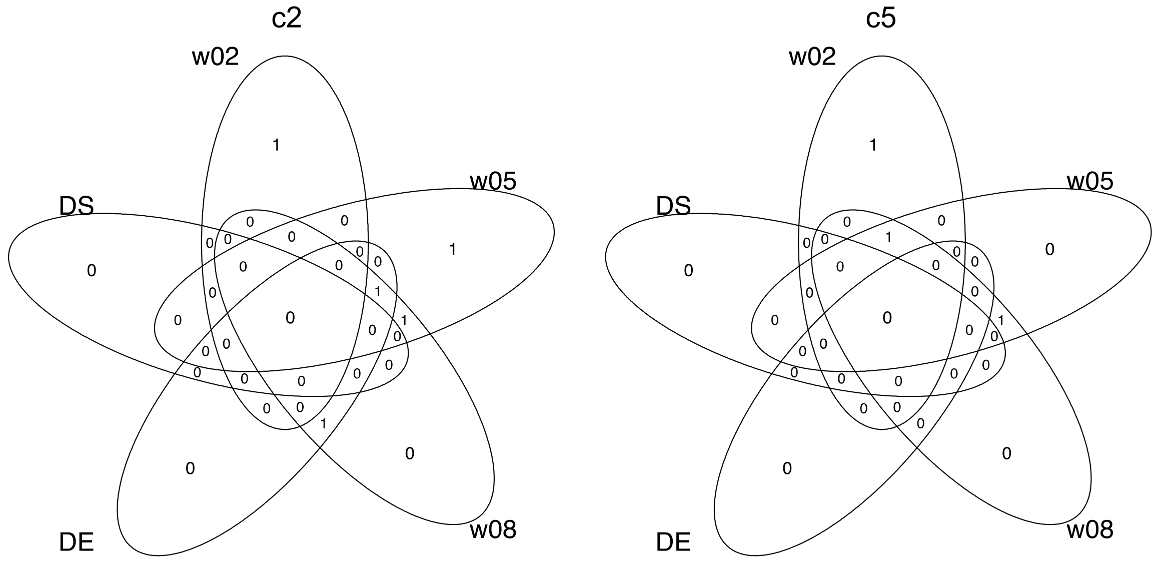

Supplementary Figure S9. Venn diagrams of significant c2 & c5 gene sets with different α on the BA46 data set at FDR 1% with linear combination strategy. DS – DS-only GSEA; DE – DE-only GSEA; w01 – weight α=0.1 and so on so forth.

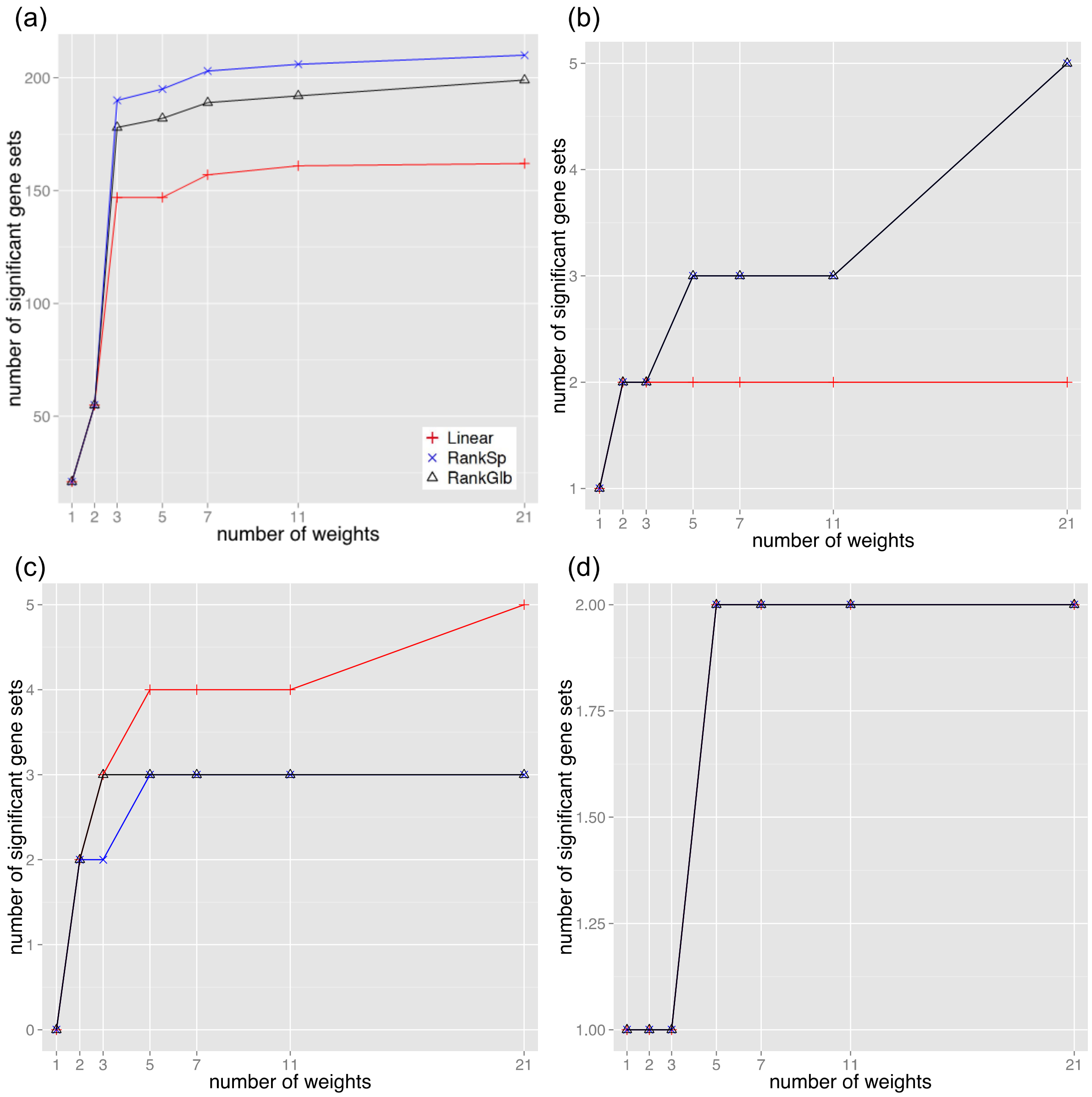

Supplementary Figure S10. Saturation plots of weights on gene set category c1. Shown are the numbers of unique gene sets detected by different number of weights indicated in x-axis. (a) is for the artificial data sets; (b) cancer; (c) BA46; (d) BA22. From the fewest to the most number of weights, we gradually included the following weights in the order of (1 – DE-only, 0 – DS-only, 0.5, 0.1, 0.9, 0.3, 0.7, 0.2, 0.4, 0.6, 0.8, 0.05, 0.15, 0.25, 0.35, 0.45, 0.55, 0.65, 0.75, 0.85, 0.95).

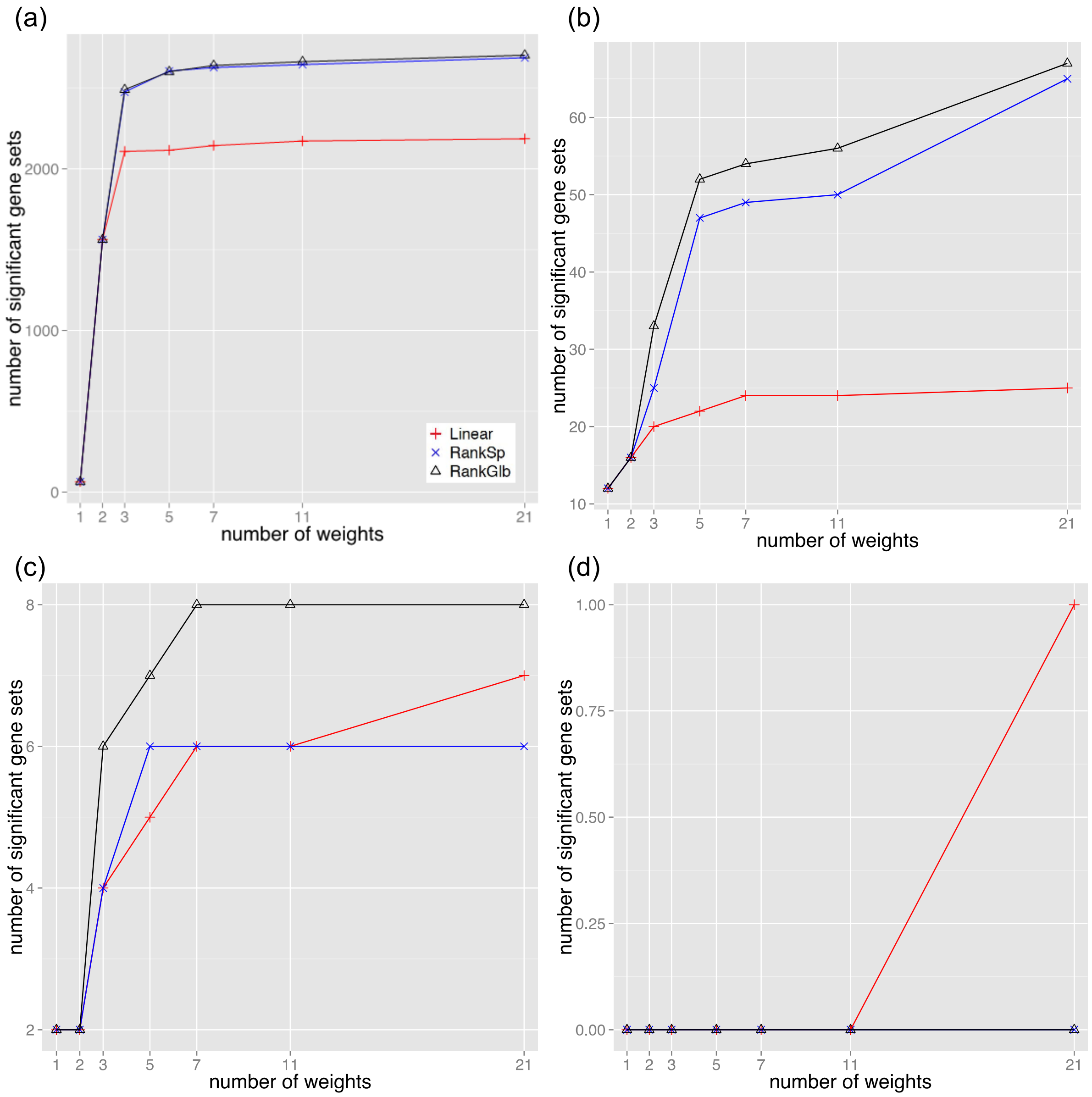

Supplementary Figure S11 Saturation plots of weights on gene set category c2. Shown are the numbers of unique gene sets detected by different number of weights indicated in x-axis. (a) is for the artificial data sets; (b) cancer; (c) BA46; (d) BA22. From the fewest to the most number of weights, we gradually included the following weights in the order of (1 – DE-only, 0 – DS-only, 0.5, 0.1, 0.9, 0.3, 0.7, 0.2, 0.4, 0.6, 0.8, 0.05, 0.15, 0.25, 0.35, 0.45, 0.55, 0.65, 0.75, 0.85, 0.95).

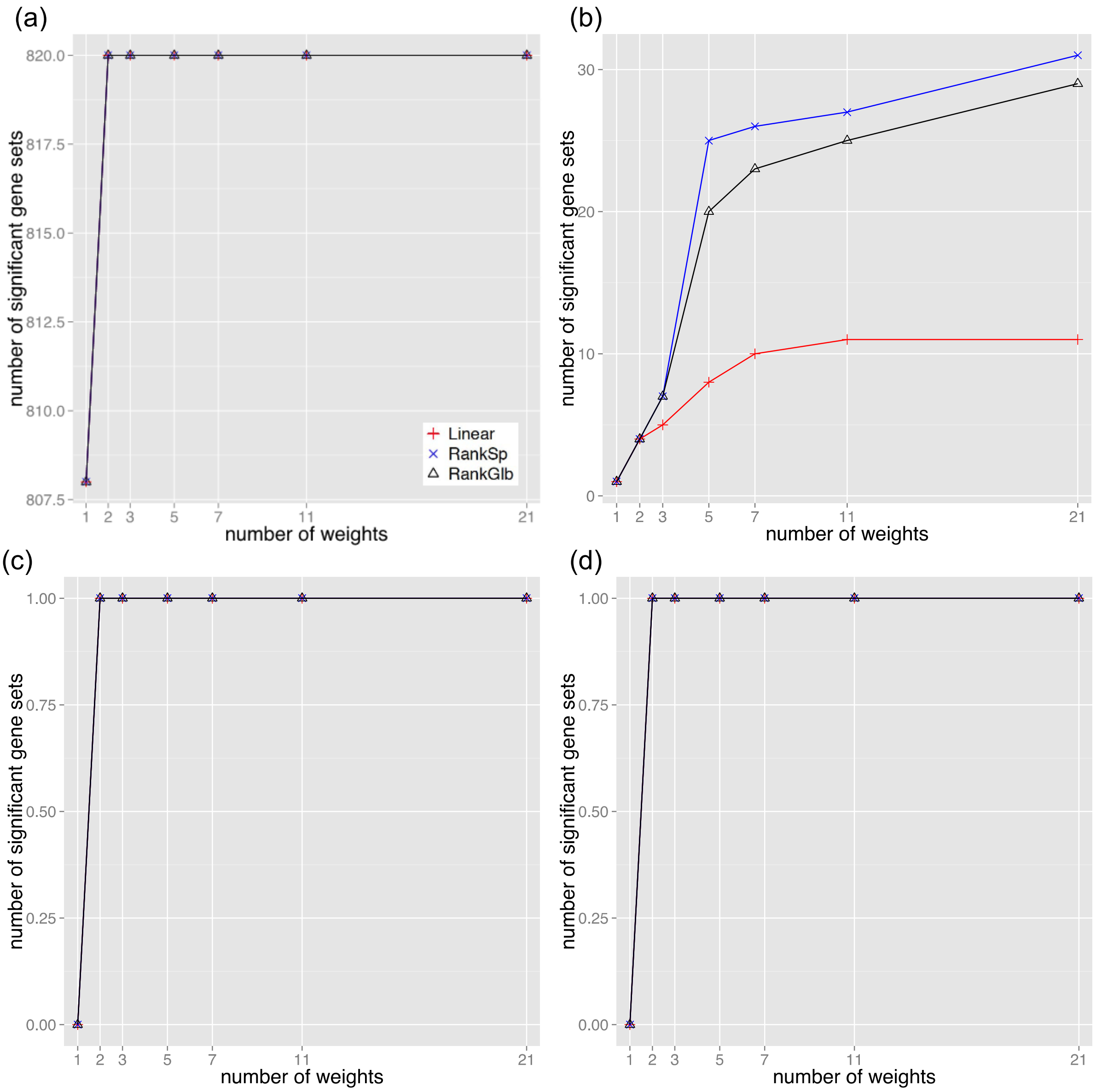

Supplementary Figure S12. Saturation plots of weights on gene set category c3. Shown are the numbers of unique gene sets detected by different number of weights indicated in x-axis. (a) is for the artificial data sets; (b) cancer; (c) BA46; (d) BA22. From the fewest to the most number of weights, we gradually included the following weights in the order of (1 – DE-only, 0 – DS-only, 0.5, 0.1, 0.9, 0.3, 0.7, 0.2, 0.4, 0.6, 0.8, 0.05, 0.15, 0.25, 0.35, 0.45, 0.55, 0.65, 0.75, 0.85, 0.95).

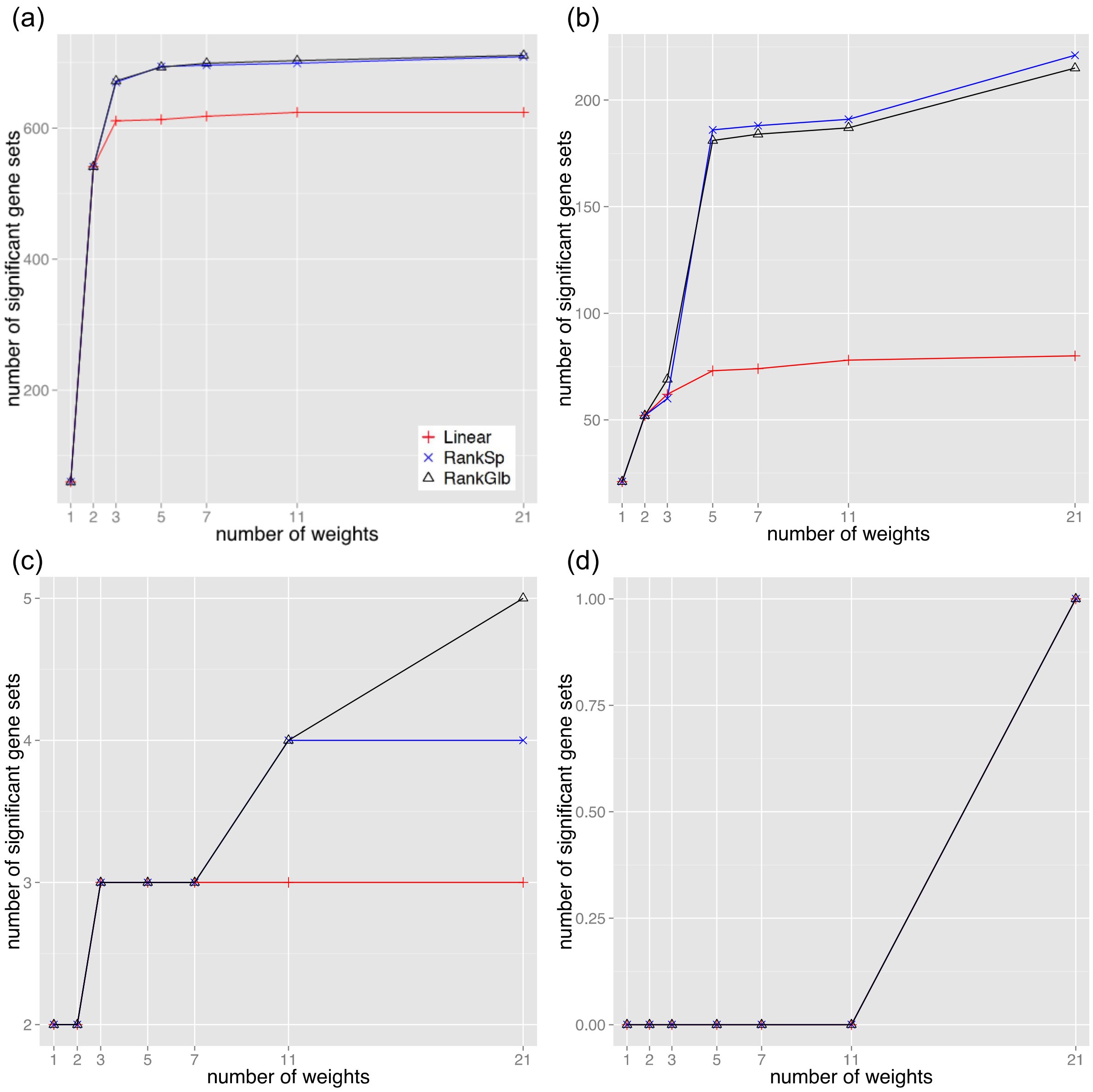

Supplementary Figure S13. Saturation plots of weights on gene set category c4. Shown are the numbers of unique gene sets detected by different number of weights indicated in x-axis. (a) is for the artificial data sets; (b) cancer; (c) BA46; (d) BA22. From the fewest to the most number of weights, we gradually included the following weights in the order of (1 – DE-only, 0 – DS-only, 0.5, 0.1, 0.9, 0.3, 0.7, 0.2, 0.4, 0.6, 0.8, 0.05, 0.15, 0.25, 0.35, 0.45, 0.55, 0.65, 0.75, 0.85, 0.95).

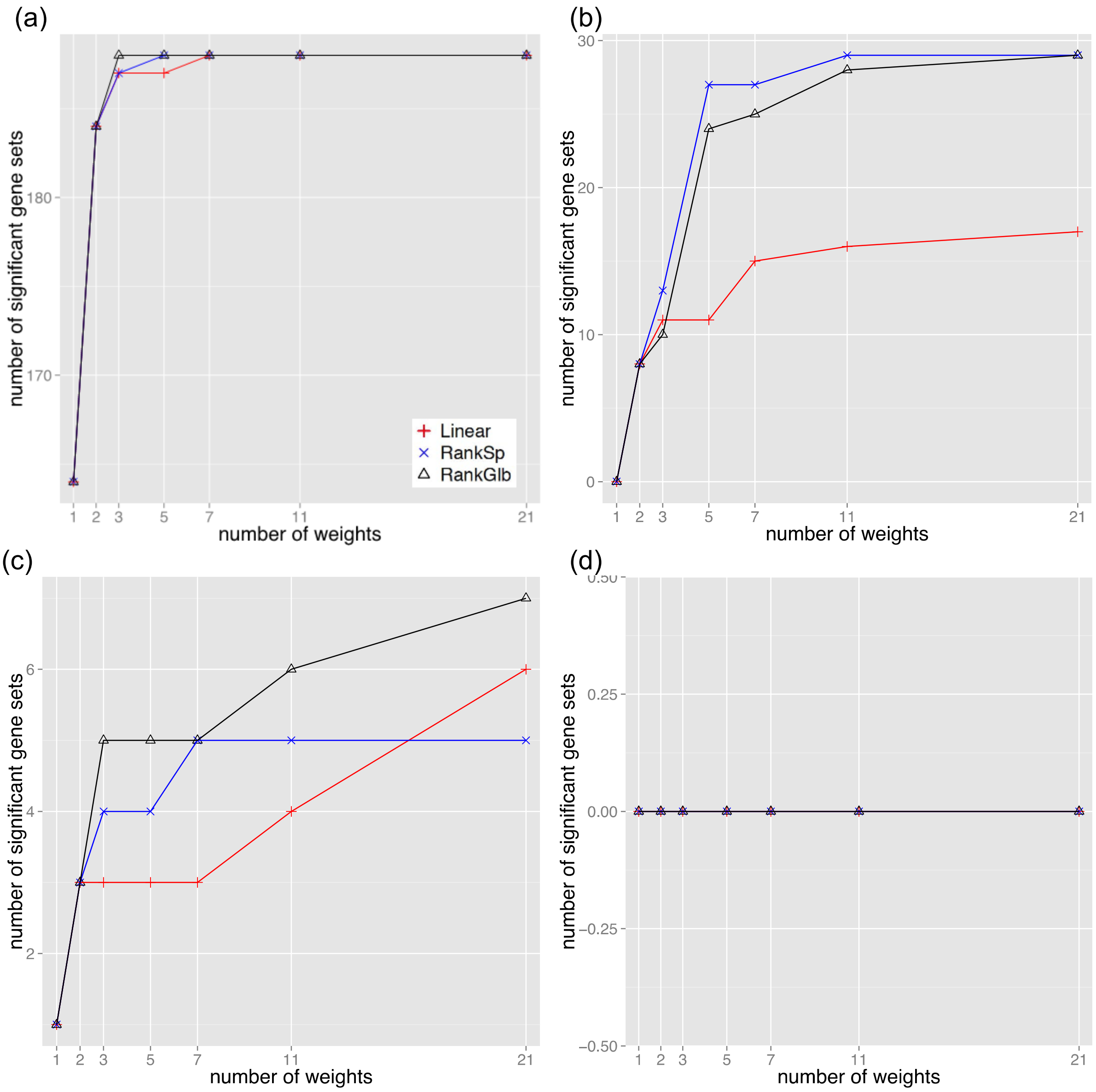

Supplementary Figure S14. Saturation plots of weights on gene set category c6. Shown are the numbers of unique gene sets detected by different number of weights indicated in x-axis. (a) is for the artificial data sets; (b) cancer; (c) BA46; (d) BA22. From the fewest to the most number of weights, we gradually included the following weights in the order of (1 – DE-only, 0 – DS-only, 0.5, 0.1, 0.9, 0.3, 0.7, 0.2, 0.4, 0.6, 0.8, 0.05, 0.15, 0.25, 0.35, 0.45, 0.55, 0.65, 0.75, 0.85, 0.95).

Section III - Supplementary Tables

Supplementary Table S1. The number of significant gene sets on the artificial data sets at FDR 1%.

	GS	DS	0.1	0.2	0.3	0.4	0.5	0.6	0.7	0.8	0.9	DE
Linear	c1	43	31	37	73	115	144	141	114	69	37	21
	c2	1550	982	1389	1656	1773	1757	1569	1247	696	261	64
	c3	817	817	820	817	816	816	817	816	815	813	808
	c4	539	439	469	483	482	465	438	335	153	72	60
	c5	647	326	479	681	766	747	675	558	354	158	66
	c6	171	171	180	184	185	185	186	183	181	174	164
RankSp	c1	43	131	151	167	169	188	182	173	156	111	21
	c2	1550	2423	2456	2441	2403	2314	2180	2038	1737	1232	64
	c3	817	820	820	819	818	818	817	817	817	815	808
	c4	539	655	646	634	621	610	571	544	483	344	60
	c5	647	1037	1048	1034	1020	971	936	867	772	576	66
	c6	171	187	188	188	187	187	187	187	187	183	164
RankGlb	c1	43	126	151	167	172	176	160	140	103	50	21
	c2	1550	2403	2474	2472	2467	2346	2140	1827	1268	496	64
	c3	817	819	819	819	817	818	817	817	816	815	808
	c4	539	648	650	648	629	604	554	461	310	94	60
	c5	647	1028	1051	1035	1027	968	888	761	553	270	66
	c6	171	188	188	188	188	188	187	186	182	180	164

GS: gene set category; DS: DS-only GSEA; DE: DE-only GSEA; 0.1,…,0.9: weights α.

Supplementary Table S2. The number of significant gene sets on the cancer data sets at FDR 1%.

	GS	DS	0.1	0.2	0.3	0.4	0.5	0.6	0.7	0.8	0.9	DE
Linear	c1	1	0	0	0	0	0	0	1	1	1	1
	c2	4	3	4	10	14	16	16	16	14	12	12
	c3	3	6	5	6	1	1	3	1	0	1	1
	c4	32	42	45	40	32	31	24	21	23	19	21
	c5	11	11	20	14	13	9	6	2	2	1	1
	c6	8	7	9	11	8	3	2	1	1	0	0
RankSp	c1	1	2	1	0	0	0	0	0	0	0	1
	c2	4	36	29	27	24	20	15	15	17	16	12
	c3	3	23	13	8	8	3	3	2	0	0	1
	c4	32	182	150	103	57	37	29	24	27	23	21
	c5	11	55	36	34	21	14	9	5	3	1	1
	c6	8	24	19	16	12	9	6	2	2	1	0
RankGlb	c1	1	2	1	0	0	0	0	0	0	0	1
	c2	4	37	33	29	35	29	18	16	14	12	12
	c3	3	18	11	10	9	3	3	1	0	0	1
	c4	32	177	153	112	68	46	33	22	21	18	21
	c5	11	50	40	37	24	17	10	5	3	1	1
	c6	8	23	20	16	11	4	1	1	0	0	0

GS: gene set category; DS: DS-only GSEA; DE: DE-only GSEA; 0.1,…,0.9: weights α.

Supplementary Table S3. The number of significant gene sets on the BA46 data sets at FDR 1%.

	GS	DS	0.1	0.2	0.3	0.4	0.5	0.6	0.7	0.8	0.9	DE
Linear	c1	2	2	2	0	1	1	1	1	1	0	0
	c2	0	1	1	2	3	3	2	4	3	2	2
	c3	1	0	0	0	0	0	0	0	0	0	0
	c4	0	0	0	0	0	2	2	1	1	1	2
	c5	0	2	2	2	2	2	2	2	2	1	0
	c6	2	1	1	0	2	1	1	1	1	1	1
RankSp	c1	2	1	1	0	0	0	1	1	1	1	0
	c2	0	0	0	2	2	3	2	2	2	4	2
	c3	1	0	0	0	0	0	0	0	0	0	0
	c4	0	0	0	0	1	2	1	1	2	1	2
	c5	0	1	2	2	2	2	2	2	2	3	0
	c6	2	2	1	1	2	2	2	2	1	1	1
RankGlb	c1	2	1	1	2	0	1	1	1	0	0	0
	c2	0	0	1	2	5	5	4	5	3	3	2
	c3	1	0	0	0	0	0	0	0	0	0	0
	c4	0	0	0	0	2	2	2	1	1	1	2
	c5	0	1	2	3	2	3	2	2	2	1	0
	c6	2	2	1	2	3	3	2	1	1	1	1

GS: gene set category; DS: DS-only GSEA; DE: DE-only GSEA; 0.1,…,0.9: weights α.

Supplementary Table S4. The number of significant gene sets on the BA22 data sets at FDR 1%.

	GS	DS	0.1	0.2	0.3	0.4	0.5	0.6	0.7	0.8	0.9	DE
Linear	c1	0	1	0	1	1	0	1	1	1	1	1
	c2	0	0	0	0	0	0	0	0	0	0	0
	c3	1	1	1	0	0	0	0	0	0	0	0
	c4	0	0	0	0	0	0	0	0	0	0	0
	c5	0	0	0	0	1	1	1	1	0	0	0
	c6	0	0	0	0	0	0	0	0	0	0	0
RankSp	c1	0	1	1	1	1	1	1	1	1	1	1
	c2	0	0	0	0	0	0	0	0	0	0	0
	c3	1	1	1	0	0	0	0	0	0	0	0
	c4	0	0	0	0	0	0	0	0	0	0	0
	c5	0	1	0	0	0	0	0	0	0	0	0
	c6	0	0	0	0	0	0	0	0	0	0	0
RankGlb	c1	0	1	1	1	1	1	1	1	1	0	1
	c2	0	0	0	0	0	0	0	0	0	0	0
	c3	1	1	1	0	0	0	0	0	0	0	0
	c4	0	0	0	0	0	0	0	0	0	0	0
	c5	0	1	0	0	0	0	1	1	1	0	0
	c6	0	0	0	0	0	0	0	0	0	0	0

GS: gene set category; DS: DS-only GSEA; DE: DE-only GSEA; 0.1,…,0.9: weights α.

Supplementary Table S5. Gene sets in c2 & c5 detected by SeqGSEA on the BA46 data at FDR 1% with linear-combination integration strategy.

Category, Parameter*	Gene Sets with Description / Analysis	#Genes	FDR*
c2, 0.1,0.2,0.3	REACTOME_OLFACTORY_SIGNALING_PATHWAY<br>Description: Genes involved in Olfactory Signaling Pathway<br>Known relevance to schizophrenia: [4-6]	120	0,0,0
c2, 0.3,0.4	YAO_TEMPORAL_RESPONSE_TO_PROGESTERONE_CLUSTER_5<br>Description: Genes co-regulated in uterus during a time course response to progesterone [PubChem=5994]: SOM cluster 5.	27	0,0
c2, 0.4,0.5,0.6,0.7	VICENT_METASTASIS_DN<br>Description: The metastasis gene signature: genes down-regulated during metastasis of NSCLC (non-small cell lung carcinoma) tumors to bone.	5	0,0,0,0
c2, 0.4,0.5,0.7,0.8	LIANG_SILENCED_BY_METHYLATION_DN<br>Description: Genes down-regulated in LD419 cells (fibroblast) after treatment with decitabine (5-aza-2'-deoxycytidine) [PubChem=451668].	11	0,0,0,0
c2, 0.5,0.6,0.7,0.8, 0.9,1	ROETH_TERT_TARGETS_UP<br>Description: Genes up-regulated in T lymphocytes overexpressing TERT [GeneID=7015] off a retrovirus vector.	8	0,0,0,0, 0,0
c2, 0.7,0.8,0.9,1	SA_PROGRAMMED_CELL_DEATH<br>Description: Programmed cell death, or apoptosis, eliminates damaged or unneeded cells.<br>Known relevance to schizophrenia: [7, 8]	12	0,0,0,0
c5, 0.1	TASTE_RECEPTOR_ACTIVITY<br>Analysis: Taste-blindness is highly associated with schizophrenia [9, 10], which, indicated from our results, is suspectedly caused through the regulation of alterative splicing.	13	0
c5, 0.1,0.2	SENSORY_PERCEPTION_OF_TASTE<br>Analysis: Taste-blindness is highly associated with schizophrenia [9, 10], which, indicated from our results, is suspectedly caused through the regulation of alterative splicing.	8	0,0
c5, 0.2,0.3,0.4,0.5, 0.6,0.7,0.8	POSITIVE_REGULATION_OF_ANGIOGENESIS<br>Analysis: Literature has shown that the failure of angiogenesis damages neurogenesis, particularly in neural structure; thus the genes involved in angiogenesis may also be important for schizophrenia [11]	9	0,0,0,0, 0,0,0
c5, 0.3,0.4	ICOSANOID_METABOLIC_PROCESS<br>Analysis: The icosanoid play important roles in neural function including synaptic plasticity, resolution of and anti-inflammatory and neuroprotective bioactivity [12]. Studies have reported that the abnormality of icosanoid is associated with schizophrenia [13].	15	0,0
c5, 0.5,0.6,0.7,0.8, 0.9	GABA_RECEPTOR_ACTIVITY<br>Analysis: Several lines of evidence have indicated that GABA deficits may contribute to the pathogenesis of schizophrenia [14-16].	11	0,0,0,0, 0

* If more than one values for parameter α, the gene set is simultaneously detected with different α; in such cases, more than one corresponding FDR values (separated by comma) are listed in the same order of listed parameters.

Supplementary Table S6. Gene sets in c2 detected by SeqGSEA on the cancer data at FDR 1% with linear-combination integration strategy.

Parameter*	Gene Sets with Description	#Genes	FDR*
0.1,0.5,0.7, 0.9	REACTOME_PURINE_RIBONUCLEOSIDE_MONOPHOSPHATE_BIOSYNTHESIS<br>Genes involved in Purine ribonucleoside monophosphate biosynthesis.	11	0,0,0,0
0.1,0.3	GOLUB_ALL_VS_AML_UP<br>Up-regulated genes highly correlated with acute lymphoblastic leukemia (ALL) vs acute myeloid leukemia (AML).	19	0,0

	NICK_RESPONSE_TO_PROC_TREATMENT_UP<br>Genes changed in neutrophils upon treatment with activated protein C (PROC) [GeneID=5624] of pulmonary inflammation induced by bacterial lipopolysaccharide (LPS)	5	0,0
0.1	NAGY_PCAF_COMPONENTS_HUMAN<br>Composition of the 2 MDa human PCAF complex.	5	0
	MENSSEN_MYC_TARGETS<br>Genes up-regulated by adenoviral expression of c-MYC [GeneID=4609] in HUVEC cells (umbilical vein endothelium).	16	0
0.3,0.5,0.7, 0.9	KEGG_RIBOSOME<br>Ribosome	87	0,0,0,0
	REACTOME_FORMATION_OF_A_POOL_OF_FREE_40S_SUBUNITS<br>Genes involved in Formation of a pool of free 40S subunits	94	0,0,0,0
	REACTOME_FORMATION_OF_THE_TERNARY_COMPLEX_AND_SUBSEQUENTLY_THE_43S_COMPLEX<br>Binding of the methionyl-tRNA initiator to the active eIF2:GTP complex results in the formation of the ternary complex. Subsequently, this Met-tRNAi:eIF2:GTP (ternary) complex binds to the complex formed by the 40S subunit, eIF3 and eIF1A, to form the 43S complex.	49	0,0,0,0
	REACTOME_GTP_HYDROLYSIS_AND_JOINING_OF_THE_60S_RIBOSOMAL_SUBUNIT<br>Genes involved in GTP hydrolysis and joining of the 60S ribosomal subunit	105	0,0,0,0
	REACTOME_INFLUENZA_VIRAL_RNA_TRANSCRIPTION_AND_REPLICATION<br>Genes involved in Influenza Viral RNA Transcription and Replication	99	0,0,0,0
	REACTOME_PEPTIDE_CHAIN_ELONGATION<br>Genes involved in Peptide chain elongation	83	0,0,0,0
	REACTOME_TRANSLATION<br>Genes involved in Translation	118	0,0,0,0
	REACTOME_TRANSLATION_INITIATION_COMPLEX_FORMATION<br>Genes involved in Translation initiation complex formation	56	0,0,0,0
	REACTOME_VIRAL_MRNA_TRANSLATION<br>Genes involved in Viral mRNA Translation	83	0,0,0,0
0.3,0.5,0.7	TIEN_INTESTINE_PROBIOTICS_6HR_UP<br>Genes up-regulated in Caco-2 cells (intestinal epithelium) after coculture with the probiotic bacteria L. casei for 6h.	57	0
0.5,0.7	TOMLINS_PROSTATE_CANCER_UP<br>Genes up-regulated in prostate cancer vs benign prostate tissue, based on a meta-analysis of five gene expression profiling studies.	34	0,0
	REACTOME_INFLUENZA_LIFE_CYCLE<br>Genes involved in Influenza Life Cycle	136	0,0
	REACTOME_REGULATION_OF_GENE_EXPRESSION_IN_BETA_CELLS<br>Genes involved in Regulation of gene expression in beta cells	98	0,0
0.5	CHNG_MULTIPLE_MYELOMA_HYPERPLOID_UP<br>Protein biosynthesis, transport or catabolism genes up-regulated in hyperploid multiple myeloma (MM) compared to the non-hyperploid MM samples.	54	0
	REACTOME_FORMATION_OF_TUBULIN_FOLDING_INTERMEDIATES_BY_CCT_TRIC<br>Genes involved in Formation of tubulin folding intermediates by CCT/TriC	19	0
	REACTOME_METABOLISM_OF_PROTEINS<br>Genes involved in Metabolism of proteins	209	0
	REACTOME_ASSOCIATION_OF_TRIC_CCT_WITH_TARGET_PROTEINS_DURING_BIOSYNTHESIS<br>Genes involved in Association of TriC/CCT with target proteins during biosynthesis	29	0

* If more than one values for parameter α, the gene set is simultaneously detected with different α; in such cases, more than one corresponding FDR values (separated by comma) are listed in the same order of listed parameters.

Analysis: Most detected gene sets are related to ribosome production and structure, protein translation, viral synthesis, etc. Many studies have shown that an up-regulation of ribosome biogenesis and deregulation of protein biosynthesis are correlated with cancer [17, 18]. Evidence also has shown that Myxovirus (influenza virus) resistance 1 (MX1) is down regulated in prostate carcinomas [19], which may raise the synthesis of viral functions in the prostate cancer cells. The detected gene sets also include known up-regulated genes in prostate cancer [20].

Supplementary Table S7. Gene sets in c5 detected by SeqGSEA on the cancer data at FDR 1% with linear-combination integration strategy.

Parameter*	Gene Sets with Description	#Genes	FDR*
0.1,0.3,0.5, 0.7	EUKARYOTIC_TRANSLATION_INITIATION_FACTOR_3_COMPLEX<br>Genes annotated by the GO term GO:0005852. A complex of several polypeptides that plays at least two important roles in protein synthesis: First, eIF3 binds to the 40S ribosome and facilitates loading of the Met-tRNA/eIF2.GTP ternary complex to form the 43S preinitiation complex. Subsequently, eIF3 apparently assists eIF4 in recruiting mRNAs to the 43S complex.	10	0,0,0,0
	TRANSLATIONAL_INITIATION<br>Genes annotated by the GO term GO:0006413. The process preceding formation of the peptide bond between the first two amino acids of a protein. This includes the formation of a complex of the ribosome, mRNA, and an initiation complex that contains the first aminoacyl-tRNA.	38	0,0,0,0
	TRANSLATION_INITIATION_FACTOR_ACTIVITY<br>Genes annotated by the GO term GO:0003743. Functions in the initiation of ribosome-mediated translation of mRNA into a polypeptide.	24	0,0,0,0
0.1,0.3,0.5	RIBOSOME_BIOGENESIS_AND_ASSEMBLY<br>Genes annotated by the GO term GO:0042254. The process of the formation of the constituents of the ribosome subunits, their assembly, and their transport to the sites of protein synthesis.	14	0,0,0
0.1,0.3	RNA_ELONGATION<br>Genes annotated by the GO term GO:0006354. The extension of an RNA molecule after transcription initiation by the addition of ribonucleotides catalyzed by an RNA polymerase.	10	0,0
	RESPONSE_TO_HEAT<br>Genes annotated by the GO term GO:0009408. A change in state or activity of a cell or an organism (in terms of movement, secretion, enzyme production, gene expression, etc.) as a result of a heat stimulus, a temperature stimulus above the optimal temperature for that organism.	10	0,0
	OXIDOREDUCTASE_ACTIVITY_ACTING_ON_PEROXIDE_AS_ACCEPTOR<br>Genes annotated by the GO term GO:0016684. Catalysis of an oxidation-reduction (redox) reaction in which the peroxide group acts as a hydrogen or electron acceptor.	11	0,0
	LIGASE_ACTIVITY_FORMING_CARBON_OXYGEN_BONDS<br>Genes annotated by the GO term GO:0016875. Catalysis of the ligation of two substances via a carbon-oxygen bond with concomitant breakage of a diphosphate linkage, usually in a nucleoside triphosphate.	13	0,0
	GLUTATHIONE_TRANSFERASE_ACTIVITY<br>Genes annotated by the GO term GO:0004364. Catalysis of the reaction: R-X + glutathione = H-X + R-S-glutathione. R may be an aliphatic, aromatic or heterocyclic group; X may be a sulfate, nitrile or halide group.	14	0,0

	ANTIOXIDANT_ACTIVITY<br>Genes annotated by the GO term GO:0016209. Inhibition of the reactions brought about by dioxygen (O2) or peroxides. Usually the antioxidant is effective because it can itself be more easily oxidized than the substance protected. The term is often applied to components that can trap free radicals, thereby breaking the chain reaction that normally leads to extensive biological damage.	16	0,0
0.1	PROTEASOME_COMPLEX<br>Genes annotated by the GO term GO:0000502. A large multisubunit complex which catalyzes protein degradation. This complex consists of the barrel shaped proteasome core complex and one or two associated proteins or complexes that act in regulating entry into or exit from the core.	23	0
	PROTEIN_TARGETING_TO_MITOCHONDRION<br>Genes annotated by the GO term GO:0006626. The process of directing proteins towards and into the mitochondrion, mediated by mitochondrial proteins that recognize signals contained within the imported protein.	10	0
	NUCLEOTIDE_SUGAR_METABOLIC_PROCESS<br>Genes annotated by the GO term GO:0009225. The chemical reactions and pathways involving nucleotide-sugars, any nucleotide in which the distal phosphoric residue of a nucleoside 5'-diphosphate is in glycosidic linkage with a monosaccharide or monosaccharide derivative.	9	0
	CHAPERONE_BINDING<br>Genes annotated by the GO term GO:0051087. Interacting selectively with a chaperone protein, a class of proteins that bind to nascent or unfolded polypeptides and ensure correct folding or transport.	12	0
0.3,0.5,0.7, 0.9	STRUCTURAL_CONSTITUENT_OF_RIBOSOME<br>Genes annotated by the GO term GO:0003735. The action of a molecule that contributes to the structural integrity of the ribosome.	79	0,0,0,0
0.3	VESICLE_COAT<br>Genes annotated by the GO term GO:0030120. A membrane coat found on a coated vesicle.	14	0
	INTEGRAL_TO_GOLGI_MEMBRANE<br>Genes annotated by the GO term GO:0030173. Located such that some or all of the gene product itself penetrates at least one phospholipid bilayer of the Golgi complex membrane. May also refer to the state of being buried in the bilayer with no exposure outside the bilayer.	10	0
	COATED_VESICLE_MEMBRANE<br>Genes annotated by the GO term GO:0030662. The lipid bilayer surrounding a coated vesicle.	14	0
	MEMBRANE_COAT<br>Genes annotated by the GO term GO:0030117. Any of several different proteinaceous coats that can associate with membranes. Membrane coats include those formed by clathrin plus an adaptor complex, the COPI and COPII complexes, and possibly others. They are found associated with membranes on many vesicles as well as other membrane features such as pits and perhaps tubules.	15	0
	COATED_MEMBRANE<br>Genes annotated by the GO term GO:0048475. A single or double lipid bilayer with any of several different proteinaceous coats that can associate with membranes. Membrane coats include those formed by clathrin plus an adaptor complex, the COPI and COPII complexes.	15	0
	INTRA_GOLGI_VESICLE_MEDIATED_TRANSPORT<br>Genes annotated by the GO term GO:0006891. The directed movement of substances within the Golgi, mediated by small transport vesicles. These either fuse with the cis-Golgi or with each other to form the membrane stacks known as the cis-Golgi reticulum (network).	12	0
	RRNA_PROCESSING<br>Genes annotated by the GO term GO:0006364. Any process involved in the conversion of a primary ribosomal RNA (rRNA) transcript into one or more mature rRNA molecules.	11	0

	RRNA_METABOLIC_PROCESS<br>Genes annotated by the GO term GO:0016072. The chemical reactions and pathways involving rRNA, ribosomal RNA, a structural constituent of ribosomes.	12	0
	TRANSLATION_REGULATOR_ACTIVITY<br>Genes annotated by the GO term GO:0045182. Any substance involved in the initiation, activation, perpetuation, repression or termination of polypeptide synthesis at the ribosome.	39	0
	TRANSLATION_FACTOR_ACTIVITY_NUCLEIC_ACID_BINDING<br>Genes annotated by the GO term GO:0008135. Functions during translation by binding nucleic acids during polypeptide synthesis at the ribosome.	37	0
0.5	REGULATION_OF_TRANSLATIONAL_INITIATION<br>Genes annotated by the GO term GO:0006446. Any process that modulates the frequency, rate or extent of translational initiation.	30	0
	N_ACETYLGLUCOSAMINE_METABOLIC_PROCESS<br>Genes annotated by the GO term GO:0006044. The chemical reactions and pathways involving N-acetylglucosamine. The D isomer is a common structural unit of glycoproteins in plants, bacteria and animals; it is often the terminal sugar of an oligosaccharide moiety of a glycoprotein.	12	0
	RIBONUCLEOPROTEIN_COMPLEX_BIOGENESIS_AND_ASSEMBLY<br>Genes annotated by the GO term GO:0022613. The cellular process by which a complex containing RNA and proteins, is synthesized, aggregates, and bonds together.	71	0
	PROTEIN_RNA_COMPLEX_ASSEMBLY<br>Genes annotated by the GO term GO:0022618. The aggregation, arrangement and bonding together of proteins and RNA molecules to form a ribonucleoprotein complex.	56	0
	ENDODEOXYRIBONUCLEASE_ACTIVITY<br>Genes annotated by the GO term GO:0004520. Catalysis of the hydrolysis of ester linkages within deoxyribonucleic acid by creating internal breaks.	11	0
	BETA_TUBULIN_BINDING<br>Genes annotated by the GO term GO:0048487. Interacting selectively with the microtubule constituent protein beta-tubulin.	10	0

* If more than one values for parameter α, the gene set is simultaneously detected with different α; in such cases, more than one corresponding FDR values (separated by comma) are listed in the same order of listed parameters.

Analysis: Gene sets detected in this category also include the ones regarding ribosome and translation. Other detected gene sets of interest are: genes responding to heat, gene sets with respect to antioxidant activity, and those involved in vesicle membrane coat. It has been reported that heat treatment can lead to apoptosis in the prostate cancer cells [21], and that antioxidants are associated with a decreased risk of prostate cancer [22]. Also, a piece of indirect evidence shows the association between the vesicle coat and the prostate cancer: SEC23A plays a major role in assembly and transport of COPII vesicles [23], and SEC23A mRNA and protein are down-regulated in prostate cancer cell lines [24].

References

1. Anders S, Huber W: **Differential expression analysis for sequence count data**. *Genome Biol* 2010, **11**(10):R106.
2. Trapnell C, Hendrickson DG, Sauvageau M, Goff L, Rinn JL, Pachter L: **Differential analysis of gene regulation at transcript resolution with RNA-seq**. *Nat Biotechnol* 2012.
3. Wang W, Qin Z, Feng Z, Wang X, Zhang X: **Identifying differentially spliced genes from two groups of RNA-seq samples**. *Gene* 2012.
4. Turetsky BI, Hahn CG, Arnold SE, Moberg PJ: **Olfactory receptor neuron dysfunction in schizophrenia**. *Neuropsychopharmacology* 2009, **34**(3):767-774.

5. Turetsky BI, Hahn CG, Borgmann-Winter K, Moberg PJ: **Scents and nonsense: olfactory dysfunction in schizophrenia**. *Schizophr Bull* 2009, **35**(6):1117-1131.
6. Nguyen AD, Shenton ME, Levitt JJ: **Olfactory dysfunction in schizophrenia: a review of neuroanatomy and psychophysiological measurements**. *Harv Rev Psychiatry* 2010, **18**(5):279-292.
7. Catts VS, Catts SV, McGrath JJ, Feron F, McLean D, Coulson EJ, Lutze-Mann LH: **Apoptosis and schizophrenia: a pilot study based on dermal fibroblast cell lines**. *Schizophr Res* 2006, **84**(1):20-28.
8. Jarskog LF: **Apoptosis in schizophrenia: pathophysiologic and therapeutic considerations**. *Curr Opin Psychiatry* 2006, **19**(3):307-312.
9. Moberg PJ, McGue C, Kanes SJ, Roalf DR, Balderston CC, Gur RE, Kohler CG, Turetsky BI: **Phenylthiocarbamide (PTC) perception in patients with schizophrenia and first-degree family members: relationship to clinical symptomatology and psychophysical olfactory performance**. *Schizophr Res* 2007, **90**(1-3):221-228.
10. Moberg PJ, Li M, Kanes SJ, Gur RE, Kamath V, Turetsky BI: **Association of schizophrenia with the phenylthiocarbamide taste receptor haplotype on chromosome 7q**. *Psychiatr Genet* 2012.
11. Schmidt-Kastner R, van Os J, H WMS, Schmitz C: **Gene regulation by hypoxia and the neurodevelopmental origin of schizophrenia**. *Schizophr Res* 2006, **84**(2-3):253-271.
12. Tassoni D, Kaur G, Weisinger RS, Sinclair AJ: **The role of eicosanoids in the brain**. *Asia Pac J Clin Nutr* 2008, **17 Suppl 1**:220-228.
13. Ross BM: **Phospholipid and eicosanoid signaling disturbances in schizophrenia**. *Prostaglandins Leukot Essent Fatty Acids* 2003, **69**(6):407-412.
14. Ishikawa M, Mizukami K, Iwakiri M, Hidaka S, Asada T: **GABAA receptor gamma subunits in the prefrontal cortex of patients with schizophrenia and bipolar disorder**. *Neuroreport* 2004, **15**(11):1809-1812.
15. Ahn K, Gil R, Seibyl J, Sewell RA, D'Souza DC: **Probing GABA receptor function in schizophrenia with iomazenil**. *Neuropsychopharmacology* 2011, **36**(3):677-683.
16. Wassef A, Baker J, Kochan LD: **GABA and schizophrenia: a review of basic science and clinical studies**. *J Clin Psychopharmacol* 2003, **23**(6):601-640.
17. Montanaro L, Trere D, Derenzini M: **Nucleolus, ribosomes, and cancer**. *Am J Pathol* 2008, **173**(2):301-310.
18. Holland EC, Sonenberg N, Pandolfi PP, Thomas G: **Signaling control of mRNA translation in cancer pathogenesis**. *Oncogene* 2004, **23**(18):3138-3144.
19. Schulz WA, Alexa A, Jung V, Hader C, Hoffmann MJ, Yamanaka M, Fritzsche S, Wlazlinski A, Muller M, Lengauer T *et al*: **Factor interaction analysis for chromosome 8 and DNA methylation alterations highlights innate immune response suppression and cytoskeletal changes in prostate cancer**. *Mol Cancer* 2007, **6**:14.
20. Tomlins SA, Mehra R, Rhodes DR, Cao X, Wang L, Dhanasekaran SM, Kalyana-Sundaram S, Wei JT, Rubin MA, Pienta KJ *et al*: **Integrative molecular concept modeling of prostate cancer progression**. *Nat Genet* 2007, **39**(1):41-51.
21. Ide H, Nakagawa T, Terado Y, Yasuda M, Kamiyama Y, Muto S, Horie S: **DNA damage response in prostate cancer cells after high-intensity focused ultrasound (HIFU) treatment**. *Anticancer Res* 2008, **28**(2A):639-643.
22. Jain MG, Hislop GT, Howe GR, Ghadirian P: **Plant foods, antioxidants, and prostate cancer risk: findings from case-control studies in Canada**. *Nutr Cancer* 1999, **34**(2):173-184.
23. Fromme JC, Orci L, Schekman R: **Coordination of COPII vesicle trafficking by Sec23**. *Trends Cell Biol* 2008, **18**(7):330-336.
24. Szczyrba J, Nolte E, Wach S, Kremmer E, Stohr R, Hartmann A, Wieland W, Wullich B, Grasser FA: **Downregulation of Sec23A protein by miRNA-375 in prostate carcinoma**. *Mol Cancer Res* 2011, **9**(6):791-800.
